# Mutation Edgotype Drives Fitness Effect in Human

**DOI:** 10.3389/fbinf.2021.690769

**Published:** 2021-08-30

**Authors:** Mohamed Ghadie, Yu Xia

**Affiliations:** Department of Bioengineering, McGill University, Montreal, QC, Canada

**Keywords:** missense mutations, mutation edgotype, protein-protein interactions, fitness effect, interactome perturbations

## Abstract

Missense mutations are known to perturb protein-protein interaction networks (known as interactome networks) in different ways. However, it remains unknown how different interactome perturbation patterns (“edgotypes”) impact organismal fitness. Here, we estimate the fitness effect of missense mutations with different interactome perturbation patterns in human, by calculating the fractions of neutral and deleterious mutations that do not disrupt PPIs (“quasi-wild-type”), or disrupt PPIs either by disrupting the binding interface (“edgetic”) or by disrupting overall protein stability (“quasi-null”). We first map pathogenic mutations and common non-pathogenic mutations onto homology-based three-dimensional structural models of proteins and protein-protein interactions in human. Next, we perform structure-based calculations to classify each mutation as either quasi-wild-type, edgetic, or quasi-null. Using our predicted as well as experimentally determined interactome perturbation patterns, we estimate that >∼40% of quasi-wild-type mutations are effectively neutral and the remaining are mostly mildly deleterious, that >∼75% of edgetic mutations are only mildly deleterious, and that up to ∼75% of quasi-null mutations may be strongly detrimental. These estimates are the first such estimates of fitness effect for different network perturbation patterns in any interactome. Our results suggest that while mutations that do not disrupt the interactome tend to be effectively neutral, the majority of human PPIs are under strong purifying selection and the stability of most human proteins is essential to human life.

## Introduction

Protein-protein interactions (PPIs) form a central component of the cellular circuitry, which determines and controls complex cellular functions, along with other biomolecular interactions (Cafarelli et al., 2017). The collective network of PPIs (known as the interactome network) has been highly utilized to advance our knowledge of protein function (Sharan et al., 2007; Yang et al., 2016), disease (Goh et al., 2007; Menche et al., 2015; Sahni et al., 2015; Vidal et al., 2011; Zhou et al., 2014), and evolution (Das et al., 2013; Ghadie et al., 2018; Qian et al., 2011; Vo et al., 2016; Zhong et al., 2016), often with the integration of protein structural information (Chen and Xia, 2019; Franzosa and Xia, 2011; Garamszegi et al., 2013; Ghadie et al., 2017; Guo et al., 2013; Kim et al., 2006; Meyer et al., 2013; Mosca et al., 2013; Mosca et al., 2015; Wang et al., 2012). Interactome networks are subject to perturbations driven by variations in protein sequence (Ghadie et al., 2018), particularly by missense mutations (Ghadie and Xia, 2019; Sahni et al., 2015). While estimates of how new missense mutations may affect fitness in human are available (27% effectively neutral, 53% mildly deleterious, and 20% strongly detrimental) (Kryukov et al., 2007), it remains unknown how the fitness effect of missense mutations varies for different patterns of interactome perturbation.

Theories in molecular evolution and population genetics (Levy et al., 2009; Lynch, 2007) as well as empirical analyses of genome-wide data (Landry et al., 2009; Levy et al., 2012; Studer et al., 2016) suggest that a significant part of the human interactome may be non-deleterious upon disruption. On the other hand, the disruption of PPIs by missense mutations is known to cause many diseases, either through the disruption of PPI binding interfaces or through the disruption of overall protein stability (Jubb et al., 2017; Stefl et al., 2013; Yates and Sternberg, 2013). Sahni et al. studied the precise interactome perturbation pattern (“edgotype”) (Sahni et al., 2013) for 197 Mendelian pathogenic mutations as well as 47 common non-pathogenic variants from healthy individuals (Sahni et al., 2015). While the vast majority (∼92%) of non-pathogenic mutations do not disrupt the interactome (“quasi-wild-type” mutations), the majority (∼57%) of pathogenic mutations disrupt the interactome, either by disrupting the binding interface (“edgetic” mutations) or by disrupting overall protein stability (“quasi-null” mutations) (Sahni et al., 2015). Using these experimentally determined mutation edgotypes as well as mutation edgotypes determined by structure-based predictions, we recently estimated that <∼20% of PPIs in the human interactome are effectively neutral upon disruption by edgetic mutations, and the remaining are at least mildly deleterious upon disruption (Ghadie and Xia, 2019). Nonetheless, the effect of quasi-null mutations and quasi-wild-type mutations on fitness in human is likely to be different. By disrupting overall protein stability, quasi-null mutations cause complex cellular and phenotypic changes that are not explainable by simple PPI disruptions. At the same time, it is possible for quasi-wild-type mutations to be deleterious if they disrupt other molecular interactions. So far, no quantitative model exists that provides estimates of the average fitness effect for quasi-wild-type and quasi-null mutations in any interactome, and how their fitness effects differ from that of edgetic mutations.

The question of how network perturbations created by genetic mutations provide the molecular link between mutations and their associated phenotypes has gained significant attention over the past decade (Yi et al., 2017), either in the context of protein-protein interactions (Sahni et al., 2015; Yi et al., 2017; Zhong, et al., 2009) or in the context of genetic interactions (Braberg et al., 2014a; Braberg et al., 2014b; Martins et al., 2015). While these studies are essential for our understanding of protein function and disease, they do not provide interactome-wide estimates of different fitness effects associated with different mutation edgotypes. Sequencing experiments have associated hundreds of genetic mutations with different disease phenotypes and behavioural disorders, including cancer (Kumar et al., 2014; Kumar et al., 2011) and autism (Iossifov et al., 2014; O'Roak et al., 2014). However, these studies focus on mutations associated with specific diseases and therefore do not represent the full range of mutation fitness effects. More importantly, these studies do not provide us with mutation edgotypes. Other studies have explored the connection between edgetic perturbation of PPIs and phenotype, but mostly in cancer (Yi et al., 2017). Computational tools such as SIFT (Sim et al., 2012) and PolyPhen-2 (Adzhubei et al., 2010) can predict the impact of individual mutations on protein function (Thusberg and Vihinen, 2009), but they also do not explicitly predict mutation edgotype and therefore are not appropriate for addressing the goal of the present study, which is to provide interactome-wide estimates of fitness effect for different mutation edgotypes. Furthermore, these tools predict phenotypes for new mutations based on sequence and structural information (Thusberg and Vihinen, 2009), whereas our study makes use of known phenotypes for existing mutations based on experimental or clinical observations, which are more accurate.

At the same time, computational studies have constructed three-dimensional (3D) structural models for PPIs (Meyer et al., 2013; Mosca et al., 2013), and other studies have mapped pathogenic mutations in human onto PPI structural models and examined their distribution relative to PPI binding interfaces (Guo et al., 2013; Mosca et al., 2015; Wang et al., 2012). Pathogenic mutations were found to be enriched at PPI interfaces, and mutations occurring at different binding interfaces were found to be associated with different disease phenotypes more likely than mutations occurring at the same interface (Guo et al., 2013; Wang et al., 2012). While these studies suggest that pathogenic mutations may be more likely to disrupt PPIs than expected by chance, with different PPI disruptions leading to different diseases, they do not predict via physics-based calculations the effect of mutations on PPI binding affinity nor on protein folding stability. Most importantly, these studies do not calculate the fitness effect distribution associated with different mutation edgotypes. A recent study has combined mutation functional information with PPI edgetic perturbations to predict network modules underlying complex disease in human (Cui et al., 2019). This study also does not provide interactome-wide estimates of fitness effect for different mutation edgotypes. Other computational methods are able to predict the effect of individual mutations on protein folding and binding free energy (∆∆G) (Li et al., 2017), including FoldX (Schymkowitz et al., 2005), mCSM-PPI2 (Rodrigues et al., 2019), DynaMut2 (Rodrigues et al., 2020) and MuPIPR (Zhou et al., 2020). While ∆∆G values predicted by these methods can be used to ultimately predict mutation edgotype, they also cannot provide interactome-wide estimates of fitness effect for different mutation edgotypes since they do not integrate mutation phenotype information into their calculations.

Nevertheless, experimental studies mapping the edgotypes of missense mutations with known phenotypic consequences are very challenging in nature and cover less than 1% of missense mutations in human, spanning a very small subset of the human interactome. Thus, there is a great need to complement these experiments with structure-based predictions of mutation edgotypes, which will allow us to assess the applicability of insights generated by experiments to the entire human interactome. A larger coverage of mutation edgotype data enabled by structure-based calculations combined with known clinical and experimental information on mutation phenotype will also allow us to estimate with high confidence the effects of different interactome perturbation patterns on organismal fitness.

Here, we estimate the fitness effect for missense mutations with different interactome perturbation patterns in human, by estimating the probabilities for quasi-wild-type mutations, edgetic mutations and quasi-null mutations to be effectively neutral, mildly deleterious or strongly detrimental. Starting with a human reference interactome mapped by experiments, we construct a human structural interactome by building three-dimensional (3D) structural models for human proteins and PPIs, using template-based homology modelling. Next, we map known pathogenic missense mutations as well as common non-pathogenic missense mutations from healthy individuals onto our human structural interactome, and perform structure-based calculations to predict whether each mutation does not disrupt the interactome (quasi-wild-type), or disrupts the interactome either by disrupting the binding interface (edgetic) or by disrupting overall protein stability (quasi-null). We integrate these results to calculate the probabilities for common mutations (assumed to be neutral) and pathogenic mutations (assumed to be mildly deleterious) to be quasi-wild-type, edgetic, or quasi-null, and then apply Bayes’ theorem to calculate the probabilities for quasi-wild-type, edgetic and quasi-null mutations to be effectively neutral, mildly deleterious or strongly detrimental. Our calculations reveal that at least ∼40% of quasi-wild-type mutations are effectively neutral, and the remaining are mostly mildly deleterious. Our calculations also reveal that at least ∼75% of edgetic mutations are mildly deleterious, and less than ∼10% may be strongly detrimental. Furthermore, we estimate that at least ∼95% of quasi-null mutations are deleterious, with as low as ∼25% being mildly deleterious and up to ∼75% being strongly detrimental. Finally, instead of using computationally predicted mutation edgotypes, we repeat our calculations using experimentally determined mutation edgotypes from Sahni et al. (Sahni et al., 2015). Our estimates of mutation fitness effect remain broadly consistent despite minimal overlap in protein space covered by computational and experimental edgotyping data.

Our estimates are the first such estimates of fitness effect for different network perturbation patterns in any interactome. Our study also provides a solid justification for the utility of interactome networks in elucidating the phenotypic consequences of genetic mutations. Finally, our study provides a quantitative foundation for further investigation of interactome network evolution.

## Results

### The Human Structural Interactome

**FIGURE 1:**
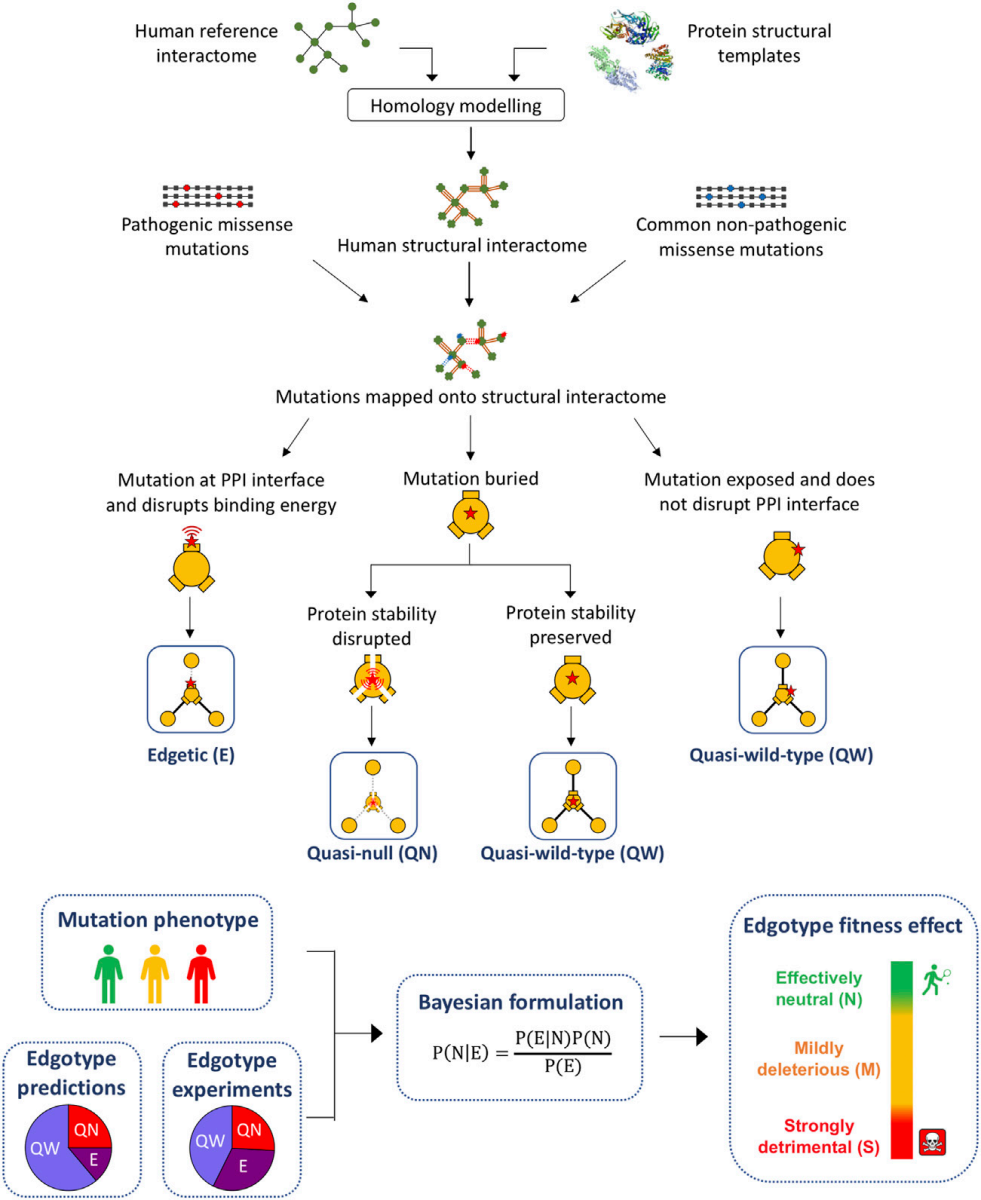
Pipeline for the computational prediction of mutation edgotypes. Computational pipeline used for the construction of the human structural interactome, prediction of mutation edgotypes and calculation of edgotype fitness effect.

We started with two high-quality human reference interactomes that were mapped by experiments: the HuRI interactome that was recently mapped using systematic yeast two-hybrid (Y2H) screens (Luck et al., 2020), and the literature-curated interactome consisting of PPIs reported by at least two independent experiments in the IntAct database (Orchard et al., 2014). From each reference interactome, we constructed a structural interactome by building 3D structural models for proteins and PPIs via homology modelling, using experimentally determined structural templates in the Protein Data Bank (PDB) (Berman et al., 2003) (Figure 1). Thus, we obtained two human structural interactomes with PPI binding interfaces annotated at the residue level: the Y2H structural interactome (Y2H-SI) consisting of 1,916 PPIs among 1,468 proteins (Supplementary Data Sheet S1A,S1B), and the literature-derived structural interactome (Lit-SI) consisting of 4,676 PPIs among 3,445 proteins (Supplementary Data Sheet S1C,S1D).

### Locating Mutations on Protein Structure

**FIGURE 2:**
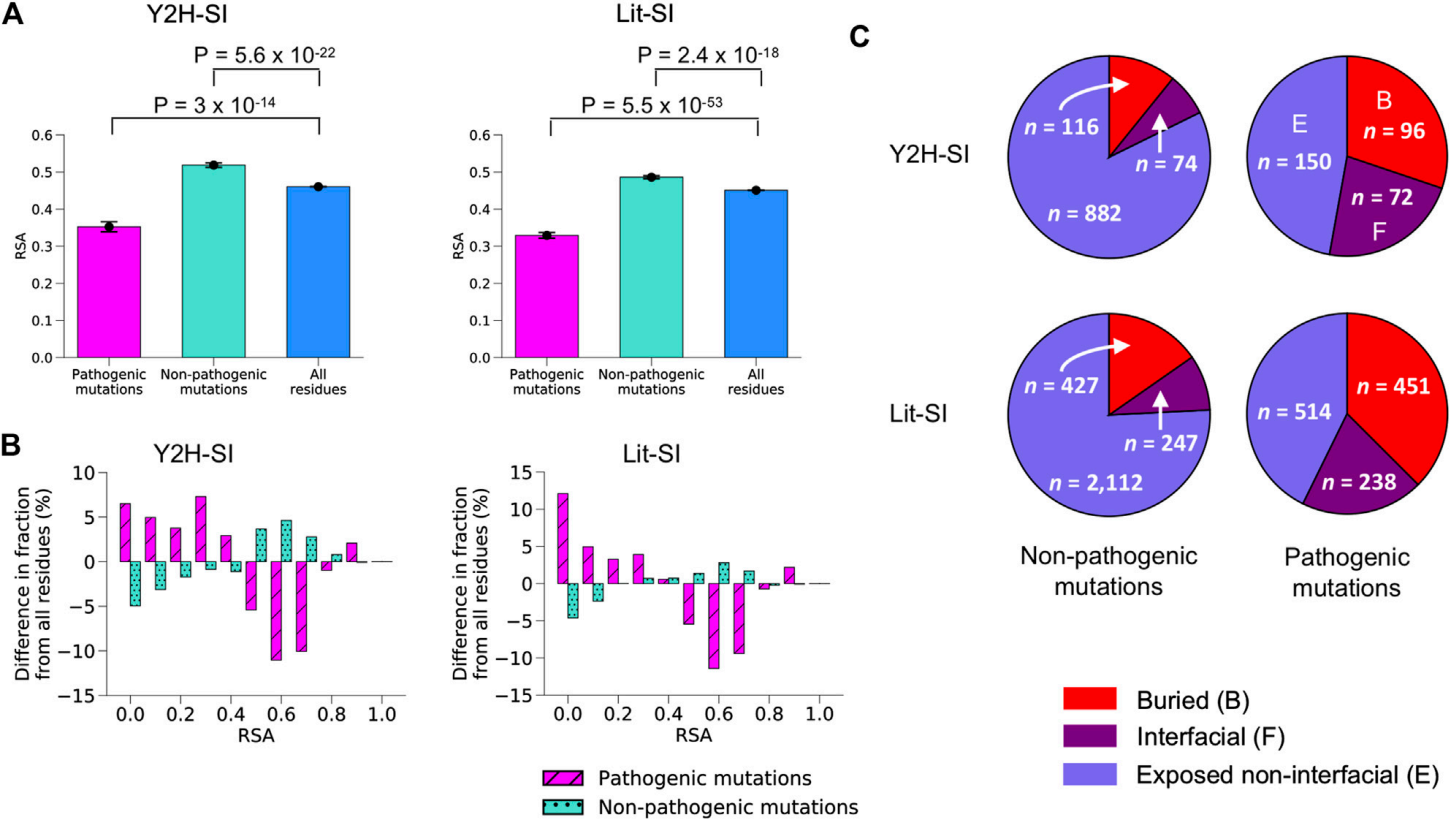
Mutation locations on protein structural models. **(A)** Average RSA for pathogenic mutations, non-pathogenic mutations and all residues in the two human structural interactomes Y2H-SI **(left)** and Lit-SI **(right)**. Error bars represent standard errors of the mean. Statistical significance was calculated using a two-sided *t*-test. **(B)** Difference in RSA distribution for pathogenic mutations and non-pathogenic mutations compared to all residues in the two human structural interactomes Y2H-SI **(left)** and Lit-SI **(right)**. **(C)** Fraction of buried mutations, interfacial mutations, and exposed non-interfacial mutations among common non-pathogenic mutations **(left)** and among pathogenic mutations **(right)** in the two human structural interactomes Y2H-SI **(top)** and Lit-SI **(bottom)**. Red slices represent buried mutations, purple slices represent interfacial mutations, and blue slices represent exposed non-interfacial mutations.

We mapped Mendelian pathogenic missense mutations from ClinVar (Landrum et al., 2016) as well as common non-pathogenic missense mutations from dbSNP (Sherry et al., 2001) onto our two human structural interactomes, Y2H-SI and Lit-SI. We obtained 1,072 common non-pathogenic mutations and 318 pathogenic mutations in Y2H-SI, and 2,786 common non-pathogenic mutations and 1,203 pathogenic mutations in Lit-SI. Next, we mapped each mutation onto the protein structural model and calculated its relative solvent accessibility (RSA). We started with mutations in Y2H-SI. We found that non-pathogenic mutations tend to have higher RSA compared to all protein residues (*p* = 5.6 × 10^−22^, two-sided *t*-test; Figures 2A,B; Supplementary Data Sheet S2A), whereas pathogenic mutations tend to have lower RSA compared to all protein residues (*p* = 3 × 10^−14^, two-sided *t*-test; Figures 2A,B; Supplementary Data Sheet S2B).

Next, we performed structure-based calculations to identify the location of each mutation on protein structure. The location of a mutation can be either at PPI binding interface, buried inside protein structure, or exposed on protein surface but not at PPI interface. If a mutation is not located at PPI interface, we predict it to be exposed on protein surface if its RSA in the protein structural model is greater than 0.25, otherwise we predict the mutation to be buried. In Y2H-SI, we found that ∼30% of pathogenic mutations are buried, ∼23% are located at PPI interfaces, and the remaining ∼47% are exposed on protein surfaces and not located at PPI interfaces (Figure 2C; Supplementary Data Sheet S2B). On the other hand, only ∼11% of non-pathogenic mutations are buried and ∼7% are located at PPI interfaces, whereas the remaining ∼82% are exposed on protein surfaces and not located at PPI interfaces (Figure 2C; Supplementary Data Sheet S2A).

We repeated the same calculations on Lit-SI. Similar to Y2H-SI, we found that non-pathogenic mutations tend to have higher RSA compared to all protein residues (*p* = 2.4 × 10^−18^, two-sided *t*-test; Figures 2A,B; Supplementary Data Sheet S2C), whereas pathogenic mutations tend to have lower RSA compared to all protein residues (*p* = 5.5 × 10^−53^, two-sided *t*-test; Figures 2A,B; Supplementary Data Sheet S2D). Also similar to Y2H-SI, we found that ∼37% of pathogenic mutations are buried, ∼20% are located at PPI interfaces, and the remaining ∼43% are exposed and not located at PPI interfaces (Figure 2C; Supplementary Data Sheet S2D). On the other hand, only ∼15% of non-pathogenic mutations are buried and ∼9% are located at PPI interfaces, whereas the remaining ∼76% are exposed and not located at PPI interfaces (Figure 2C; Supplementary Data Sheet S2C). All together, our results show that pathogenic mutations are more likely to be either buried or located at PPI interfaces than non-pathogenic mutations, suggesting that they are more likely to disrupt PPIs either by disrupting overall protein stability or by disrupting specific binding interfaces.

### Structure-Based Prediction of Mutation Edgotypes

**FIGURE 3:**
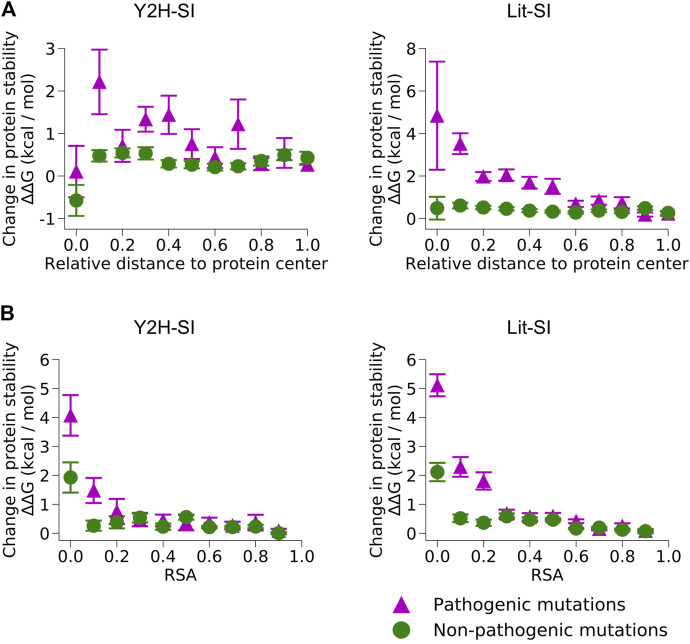
Change in protein stability upon mutation. Change in protein folding free energy (∆∆G) created by pathogenic mutations and non-pathogenic mutations in the two human structural interactomes, Y2H-SI **(left)** and Lit-SI **(right)**, in relation to **(A)** mutation distance to protein center relative to protein radius and **(B)** relative solvent accessibility (RSA) of the mutation residue.

We used our results of mutation location on protein structure to perform structure-based predictions of the edgotype for each mutation, i.e., the precise pattern of interactome perturbation as a result of each mutation (Figure 1). Mutations can be either edgetic (i.e., disrupt specific PPIs by disrupting binding interfaces), quasi-null (i.e., disrupt all PPIs by disrupting overall protein stability), or quasi-wild-type (i.e., do not disrupt any PPIs) (Sahni et al., 2015). We first predicted edgetic mutations by calculating the change in PPI binding free energy (∆∆G) caused by each mutation that is located at PPI interface using the widely known method FoldX (Schymkowitz et al., 2005) (Supplementary Figure S1A; Supplementary Data Sheet S3). We predict an interfacial mutation to be edgetic if it causes a binding ∆∆G > 0.5 kcal/mol, otherwise we predict the mutation to be non-edgetic. Next, we used FoldX to calculate the change in protein folding free energy (∆∆G) for all mutations mapped onto protein structural models (Supplementary Figure S1B; Supplementary Data Sheet S4). We found that for pathogenic mutations, while folding ∆∆G strongly correlates with both mutation RSA and mutation distance to protein center relative to protein size, it decreases significantly (<2 kcal/mol) for exposed mutations with RSA >0.25 (Figure 3). Hence, we predicted non-edgetic mutations to be quasi-null or quasi-wild-type based on the following rule: If a mutation is exposed on the surface of the protein structural model, we predict the mutation to be quasi-wild-type. On the other hand, if a mutation is buried inside the protein structural model, we predict it to be quasi-null if it causes a folding ∆∆G ≥ 2 kcal/mol, otherwise we predict it to be quasi-wild-type (Figure 1).

**FIGURE 4:**
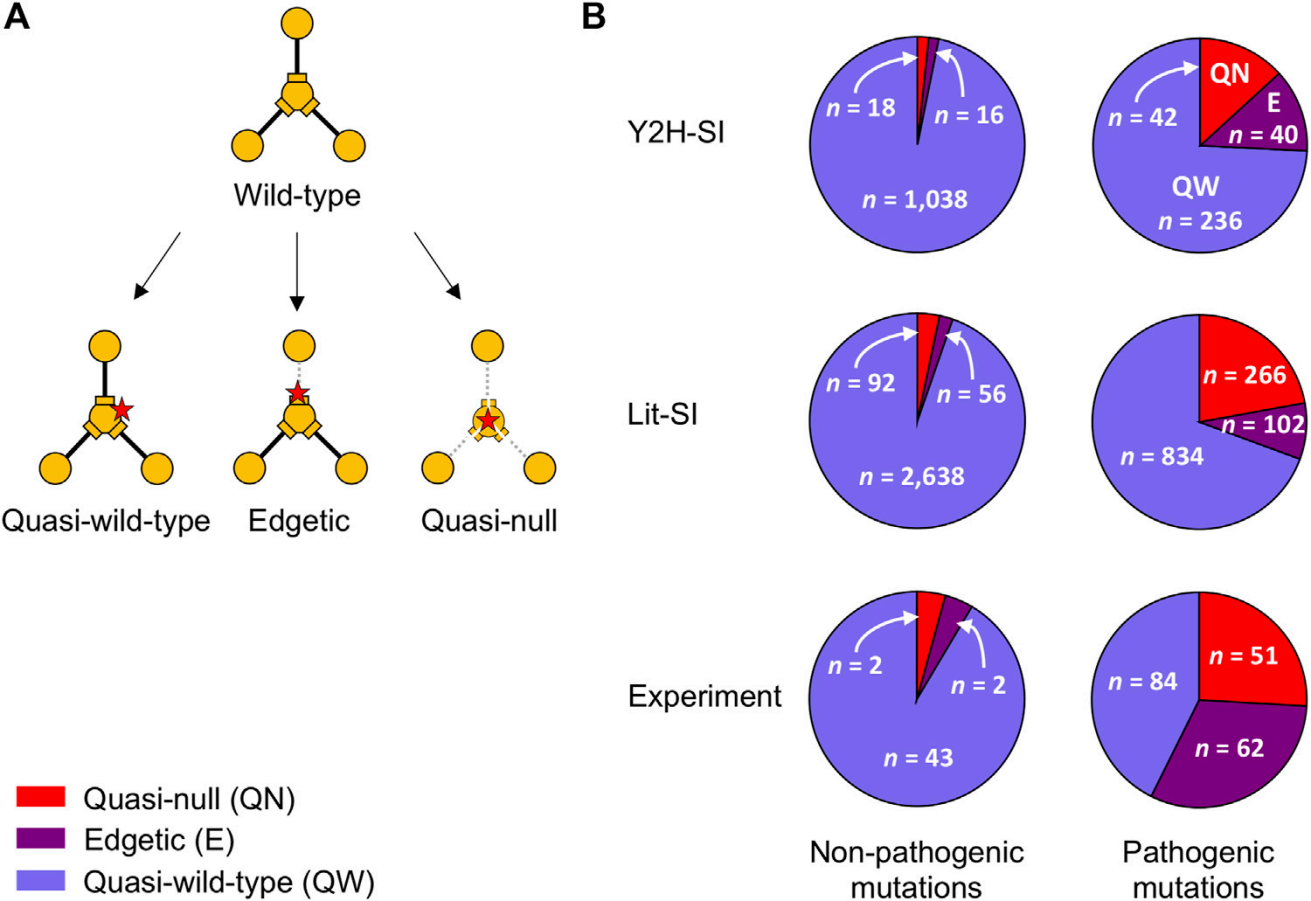
Mutation edgotypes determined by predictions and experiments. **(A)** Graphical description of quasi-wild-type mutations, edgetic mutations, and quasi-null mutations. **(B)** Fraction of quasi-wild-type mutations, edgetic mutations, and quasi-null mutations among common non-pathogenic mutations **(left)** and among pathogenic mutations **(right)**. Mutation edgotypes were obtained from structure-based predictions in the two human structural interactomes, Y2H-SI **(top)** and Lit-SI **(middle)**, and from experiments **(bottom)**. Red slices represent quasi-null mutations, purple slices represent edgetic mutations, and blue slices represent quasi-wild-type mutations.

In Y2H-SI, out of 1,072 non-pathogenic mutations, we predicted that ∼1.5% are quasi-null, ∼1.5% are edgetic, and ∼97% are quasi-wild-type (Figure 4; Supplementary Data Sheet S2A). On the other hand, out of 318 pathogenic mutations, we predicted that ∼13% are quasi-null, ∼13% are edgetic, and ∼74% are quasi-wild-type (Figure 4; Supplementary Data Sheet S2B). In Lit-SI, out of 2,786 non-pathogenic mutations, we predicted that ∼3% are quasi-null, ∼2% are edgetic, and ∼95% are quasi-wild-type (Figure 4; Supplementary Data Sheet S2C). On the other hand, out of 1,202 pathogenic mutations, we predicted that ∼22% are quasi-null, ∼9% are edgetic, and ∼69% are quasi-wild-type (Figure 4; Supplementary Data Sheet S2D).

In comparison, in the experimental study of Sahni et al., it was found that out of 47 non-pathogenic mutations, ∼4% are quasi-null, ∼4% are edgetic, and ∼92% are quasi-wild-type (Sahni et al., 2015) (Figure 4). On the other hand, it was found that out of 197 pathogenic mutations, ∼26% are quasi-null, ∼31% are edgetic, and ∼43% are quasi-wild-type (Sahni et al., 2015) (Figure 4). Thus, our computational results are consistent with experimental results in that pathogenic mutations are significantly more likely to be edgetic or quasi-null compared to non-pathogenic mutations (*p* < 10^−9^ for both computations and experiments, two-sided Fisher’s exact test).

### Fitness Effect for Quasi-wild-type, Edgetic and Quasi-Null Mutations

**FIGURE 5:**
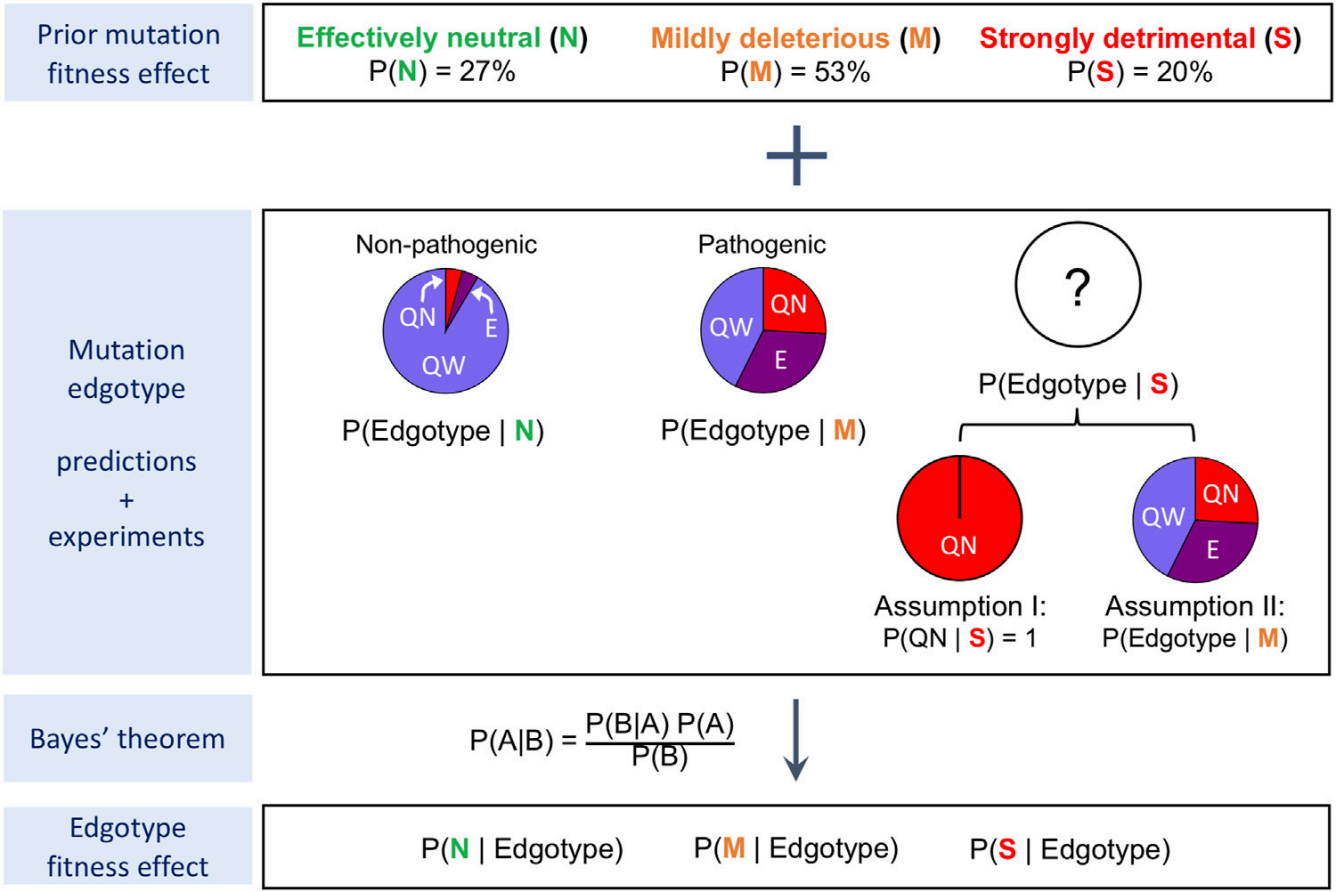
Procedure for the calculation of edgotype fitness effect. Bayesian framework used for the calculation of fitness effects for quasi-wild-type mutations, edgetic mutations and quasi-null mutations. Under Assumption I, strongly detrimental mutations are all quasi-null. Under Assumption II, strongly detrimental mutations are as likely as mildly deleterious mutations to be quasi-wild-type, edgetic or quasi-null.

We used the mutation edgotypes predicted in the previous section to estimate the fitness effect for quasi-wild-type, edgetic and quasi-null mutations by applying the Bayesian formulation we had previously developed (Ghadie et al., 2018) and describe here in the Methods section and in Figure 5. We assume that mutations are either effectively neutral (similar to synonymous mutations), mildly deleterious, or strongly detrimental (similar to nonsense mutations that introduce premature stop codons). In addition, we assume that common mutations from healthy individuals are effectively neutral, that Mendelian pathogenic mutations are mildly deleterious on average, and that strongly detrimental mutations are predominantly quasi-null (i.e., disrupt overall protein stability) rather than edgetic (Assumption I; Figure 5).

**FIGURE 6:**
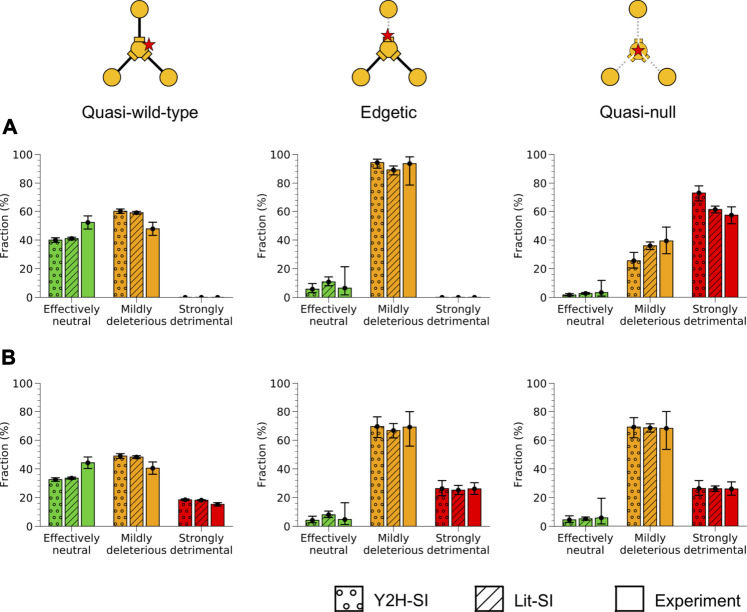
Fitness effect for different mutation edgotypes in human. Probabilities for quasi-wild-type mutations **(left)**, edgetic mutations **(middle)**, and quasi-null mutations **(right)** to be effectively neutral, mildly deleterious, or strongly detrimental in human. Probabilities were estimated from mutation edgotypes obtained by structure-based predictions in the two human structural interactomes, Y2H-SI and Lit-SI, and from mutation edgotypes obtained by experiments. **(A)** Fitness effect calculated assuming that strongly detrimental mutations are all quasi-null (Assumption I). **(B)** Fitness effect calculated assuming that strongly detrimental mutations are as likely as mildly deleterious mutations to be quasi-wild-type, edgetic or quasi-null (Assumption II). Error bars represent 95% confidence intervals.

Using our predicted mutation edgotypes in Y2H-SI, we obtained the probabilities for effectively neutral (N), mildly deleterious (M), and strongly detrimental (S) mutations to be quasi-wild-type (QW): *P* (QW|N) = 97%, *P* (QW|M) = 74%, and *P* (QW|S) ∼ = 0 assuming strongly detrimental mutations are predominantly quasi-null rather than edgetic (Figure 4). Furthermore, we obtained from (Kryukov et al., 2007) the probabilities for new missense mutations to be effectively neutral (N), mildly deleterious (M), or strongly detrimental (S): *P* (N) = 27%, *P* (M) = 53%, *P* (S) = 20%. We then integrated these numbers to calculate the probability for new missense mutations to be quasi-wild-type: *P* (QW) = *P* (QW|N)*P* (N) + *P* (QW|M)*P* (M) + *P* (QW|S)*P* (S) = 65.5%. Finally, using Bayes’ theorem *P* (A|B) = *P* (B|A)*P* (A)/*P* (B), we calculated the probabilities for quasi-wild-type mutations (QW) to be effectively neutral (N), mildly deleterious (M), or strongly detrimental (S): *P* (N|QW) = *P* (QW|N)*P* (N)/*P* (QW) = 40%, *P* (M|QW) = *P* (QW|M)*P* (M)/*P* (QW) = 60%, *P* (S|QW) = *P* (QW|S)*P* (S)/*P* (QW) = 0. Thus, we estimated that ∼40% of quasi-wild-type missense mutations in human are effectively neutral with a 95% confidence interval of ∼38–42%, and that the remaining ∼60% are mildly deleterious with a 95% confidence interval of ∼58–62% (Figure 6A; Supplementary Table S1).

Next, we repeated the same calculation using our predicted mutation edgotypes in Lit-SI (Figure 4), and estimated that ∼41% of quasi-wild-type missense mutations in human are effectively neutral with a 95% confidence interval of ∼40–42%, and that the remaining ∼59% are mildly deleterious with a 95% confidence interval of ∼58–60% (Figure 6A; Supplementary Table S1). Finally, we repeated the same calculation using the experimental mutation edgotype data from Sahni et al. (Figure 4), and estimated that ∼52% of quasi-wild-type missense mutations in human are effectively neutral with a 95% confidence interval of ∼48–57%, and that the remaining ∼48% are mildly deleterious with a 95% confidence interval of ∼43–52% (Figure 6A; Supplementary Table S1). These estimates of fitness effect for quasi-wild-type mutations obtained from predicted and experimental mutation edgotypes are broadly consistent with one another.

Following the same procedure as above, described in the Methods section and in Figure 5, we estimated the fitness effect for edgetic (E) mutations and quasi-null (QN) mutations using our predicted mutation edgotypes in Y2H-SI and Lit-SI as well as mutation edgotypes obtained from experiments (Figure 6A; Supplementary Table S1). We also assumed here that strongly detrimental (S) mutations are predominantly quasi-null, i.e., *P* (QW|S) = 0, *P* (E|S) = 0, and *P* (QN|S) = 1 (Assumption I; Figure 5). Altogether, our results reveal that >∼40% of quasi-wild-type mutations are effectively neutral and the remaining are mildly deleterious, that >∼80% of edgetic mutations are only mildly deleterious, and that as low as ∼25% of quasi-null mutations are mildly deleterious and up to ∼75% may be strongly detrimental (Figure 6A).

### Edgotype Fitness Effect Assuming Strongly Detrimental Mutations Are Similar in Edgotype to Mildly Deleterious Mutations

Our estimates of fitness effect for quasi-wild-type, edgetic and quasi-null mutations calculated above follow the assumption that strongly detrimental mutations are predominantly quasi-null rather than edgetic or quasi-wild-type (Assumption I; Figure 5). Although this is a reasonable assumption given the more radical nature of quasi-null mutations compared to other types of mutations, it is possible that some strongly detrimental mutations are edgetic or quasi-wild-type. To investigate the extent to which such cases may impact our estimate of mutation fitness effect, we repeated our calculations, this time assuming the opposite extreme but unlikely scenario that strongly detrimental (S) mutations are as likely as mildly deleterious (M) mutations to be quasi-wild-type (QW), edgetic (E) or quasi-null (QN) (Assumption II; Figure 5). In other words, instead of assuming *P* (QW|S) = 0, *P* (E|S) = 0 and *P* (QN|S) = 1 as in our previous Assumption I, we assume here in Assumption II that *P* (QW|S) = *P* (QW|M), *P* (E|S) = *P* (E|M), and *P* (QN|S) = *P* (QN|M). We repeated our calculations as in the previous section using our new Assumption II for the edgotype distribution of strongly detrimental mutations, and estimated the fitness effect for quasi-wild-type, edgetic and quasi-null mutations again (Figure 5). Our estimates of mutation fitness effect calculated from predicted mutation edgotypes as well as experimental mutation edgotypes under Assumption II are shown in Figure 6B and Supplementary Table S2.

**FIGURE 7:**
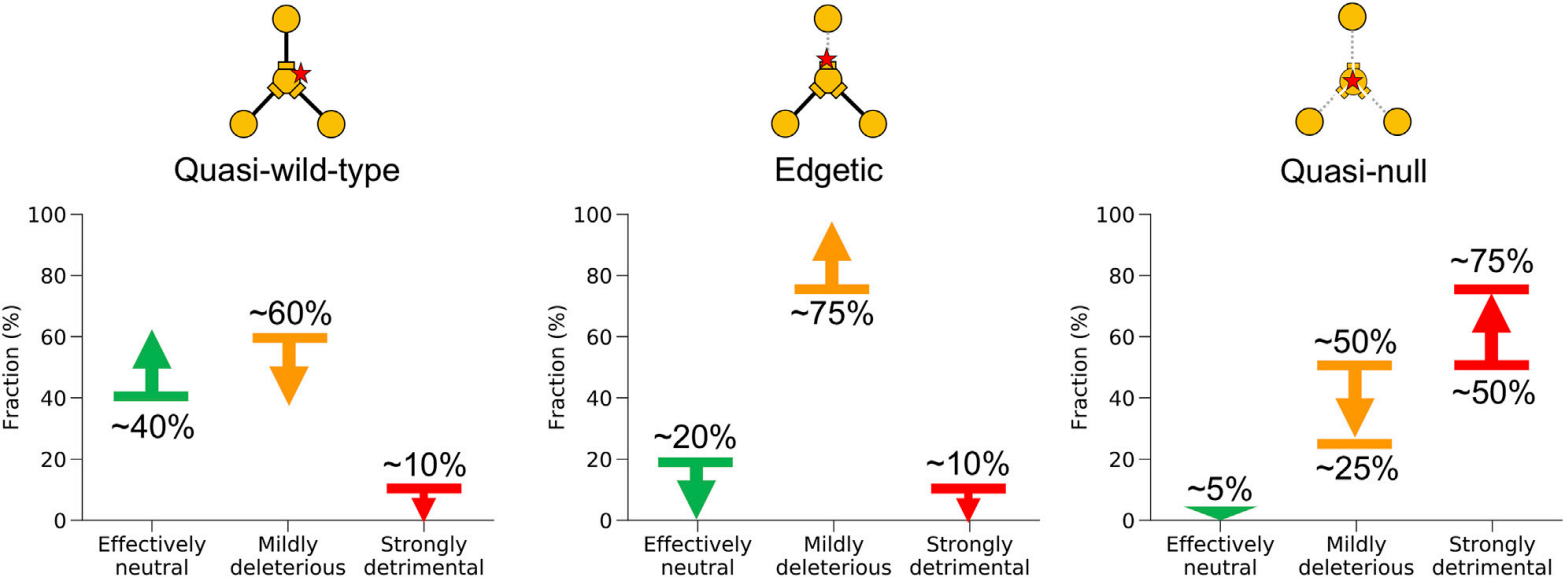
Summary of fitness effect for different mutation edgotypes in human. Probabilities for quasi-wild-type mutations, edgetic mutations, and quasi-null mutations to be effectively neutral, mildly deleterious, or strongly detrimental in human. Probabilities were obtained by merging the results from mutation fitness effect calculations under both assumptions (I) that strongly detrimental mutations are quasi-null rather than edgetic or quasi-wild-type, and (II) that strongly detrimental mutations are as likely as mildly deleterious mutations to be quasi-null, edgetic or quasi-wild-type.

Under Assumption II, the probabilities for quasi-wild-type and edgetic mutations to be strongly detrimental may reach ∼20 and ∼25%, respectively, compared to our previous estimates of ∼0 for both edgotypes obtained under Assumption I. On the other hand, the probability for quasi-null mutations to be strongly detrimental decreased from its upper limit of ∼75% under Assumption I to ∼25% under Assumption II, whereas the probability for quasi-null mutations to be mildly deleterious increased from its lower limit of ∼25% under Assumption I to ∼70% under Assumption II. All other fitness effect estimates obtained under Assumption II remain close to our estimates obtained under Assumption I. Notably, Assumptions I and II represent the two extreme scenarios for the unknown edgotype distribution associated with strongly detrimental mutations, with the real distribution being somewhere in between. Nonetheless, we believe that Assumption I is a better approximation to reality than Assumption II given the radical nature of strongly detrimental mutations. Hence, we estimate that the fitness effect for quasi-wild-type, edgetic and quasi-null mutations is somewhere between our estimate derived from Assumption I (shown in Figure 6A) and its average with our other estimate derived from Assumption II (shown in Figure 6B). We summarize these merged estimates from both assumptions in Figure 7.

## Discussion

Our estimates of mutation fitness effect (either neutral, mildly deleterious or strongly detrimental) were derived from structure-based predictions of mutation edgotypes as well as mutation edgotypes determined by experiments. Our computational predictions of mutation edgotypes were performed in two human structural interactomes, Y2H-SI and Lit-SI, that were constructed from diverse PPI datasets, HuRI and IntAct, respectively. These predictions complement experimental data as they probe different subsets of the human proteome, with <5% of computational data covered by experiments. Despite the minimal overlap in protein coverage, our estimates of fitness effect for quasi-wild-type, edgetic and quasi-null mutations derived from structure-based calculations and experiments are consistent with one another. Moreover, the HuRI dataset used in this study is much larger than the HI-II-14 dataset (Rolland et al., 2014) that was used in (Ghadie and Xia, 2019) to estimate dispensable content in the human interactome, with ∼90% of the human protein-coding genome covered by the HuRI dataset (Luck et al., 2020). Our results obtained from the HuRI dataset are consistent with our results in (Ghadie and Xia, 2019).

Genome-wide data for strongly detrimental mutations is not available. We overcome this limitation by estimating mutation fitness effect using two sets of calculations. The first set of calculations (Figure 6A) makes the assumption that strongly detrimental mutations are predominantly quasi-null rather than edgetic or quasi-wild-type (Assumption I). This assumption is very reasonable given the much more destructive nature of strongly detrimental mutations compared to mildly deleterious mutations. The second set of calculations (Figure 6B) makes the assumption that strongly detrimental mutations are as likely as mildly deleterious mutations to be quasi-null, edgetic or quasi-wild-type (Assumption II). This second relaxed assumption represents the least destructive scenario possible for strongly detrimental mutations. Although very unlikely to be true, the second assumption allows us to explore the extreme limits of mutation fitness effect in the absence of genome-wide data for strongly detrimental mutations. Thus, we estimate that the fitness effect for quasi-wild-type, edgetic and quasi-null mutations is somewhere between our estimate derived from Assumption I (shown in Figure 6A) and its average with our other estimate derived from Assumption II (shown in Figure 6B). Taking our results all together, we estimate that at least ∼40% of quasi-wild-type mutations are effectively neutral, less than ∼60% are mildly deleterious, and less than ∼10% may be strongly detrimental (Figure 7). We also estimate that at least ∼75% of edgetic mutations are mildly deleterious, and less than ∼10% may be strongly detrimental (Figure 7). Finally, we estimate that at least ∼95% of quasi-null mutations are deleterious, with as low as ∼25% being mildly deleterious and up to ∼75% being strongly detrimental (Figure 7).

PPI datasets are known to contain experimental false positives (erroneous PPIs) (Landry et al., 2013; von Mering et al., 2002; Wodak et al., 2013), which include, among others, experimental artifacts that are non-reproducible under similar experimental conditions, physical interactions that are observed *in vitro* but do not happen *in vivo*, and indirect interactions between pairs of proteins that belong to the same complex. Our goal is to estimate the fitness effects of missense mutations with different interactome perturbation patterns as defined among physical interactions that are free from these experimental errors. We have applied several measures to minimize false positive errors. First, the two human structural interactomes, Y2H-SI and Lit-SI, were constructed from experimentally determined PPIs rather than predicted PPIs. Second, Y2H-SI was derived from the high-quality HuRI dataset, which was subjected to multiple Y2H screens and other quality control measures, and is similar in quality to a gold standard dataset of literature-derived PPIs (Luck et al., 2020). Third, Lit-SI was derived from PPIs that were reported by at least two independent experiments in the literature. Fourth, our homology modelling approach that was used to construct Y2H-SI and Lit-SI enriches for true physical interactions and minimizes the occurrence of false positives, by including only PPIs for which we were able to construct homology models using experimentally determined 3D structural templates of interacting proteins in PDB.

Despite these efforts, it may be that the false positive rates of the Y2H-SI and Lit-SI datasets are non-negligible. These erroneous PPIs typically cannot discriminate between deleterious mutations and neutral mutations, since they do not physically occur in the cell. Consequently, the fitness effect probabilities for any mutation calculated in the noise portion of the interactome are independent of its edgotype. More specifically, in the false positive portion of the interactome, the posterior probabilities for quasi-wild-type mutations, edgetic mutations and quasi-null mutations to be effectively neutral (N), mildly deleterious (M) or strongly detrimental (S) are similar to the prior probabilities *P* (N), *P* (M), and *P* (S) for missense mutations to be effectively neutral, mildly deleterious or strongly detrimental, respectively. Since the error-free portion of the PPI dataset must distinguish deleterious mutations from neutral mutations better than the average performance of the entire PPI dataset, the fitness effect probabilities calculated over the error-free portion of the dataset must be even further from the prior probabilities *P* (N), *P* (M), and *P* (S) than calculated over the entire dataset. Our estimated probability for quasi-wild-type mutations to be effectively neutral (>∼40%) is higher than the prior probability for missense mutations to be effectively neutral (27%), thus our estimate represents a lower bound in the presence of experimental false positives in PPI datasets. Similarly, our estimated probability for edgetic mutations to be mildly deleterious (>∼75%) also represents a lower bound. At the same time, our estimated probabilities for quasi-wild-type mutations and edgetic mutations to be strongly detrimental (<∼10% each) are lower than the prior probability for missense mutations to be strongly detrimental (20%), thus they represent upper bounds in the presence of experimental false positives.

Notably, our estimates of fitness effect for quasi-null mutations obtained from structure-based predictions are robust to the presence of experimental false positives in the PPI dataset. This is because we predict that a non-edgetic mutation disrupts all protein interactions and thus is quasi-null if and only if it is buried inside the protein structure and significantly disrupts overall protein stability. This approach to predicting quasi-null mutations is independent of the number of protein interactions, and does not require us to predict disruption or non-disruption of each PPI individually. Thus our structure-based estimates of fitness effect for quasi-null mutations are also independent of the number of protein interactions, and are robust to the presence of false positives in the PPI dataset.

PPI datasets are also known to contain false negatives (Wodak et al., 2013). One type of false negatives are PPIs that are missing in the dataset due to incompleteness of interactome networks (Vidal, 2016). We address this limitation by considering three PPI datasets with different sizes: the high-quality dataset of HuRI where all possible pairs of proteins were tested for interaction, the larger dataset of literature-curated PPIs in IntAct, as well as the experimental dataset of Sahni et al. Another type of false negatives are true physical interactions that occur *in vivo* but are not detected *in vitro* due to experimental biases (Wodak et al., 2013). We address these biases by considering PPIs that were mapped using diverse experimental methods, including among others, Y2H experiments used to map both the HuRI dataset and part of the IntAct dataset, and affinity capture experiments used to map other parts of the IntAct dataset.

Besides these measures, false negatives may only impact our estimates by misclassifying an edgetic mutation located on protein surface as quasi-wild-type, whereas our predictions of quasi-null mutations are independent of the number of protein interactions and are robust to the presence of false negatives. Nonetheless, pathogenic mutations are known to be enriched at PPI interfaces compared to non-pathogenic mutations (Sahni et al., 2015; Wang et al., 2012). Thus, an edgetic pathogenic mutation is more likely to be misclassified as quasi-wild-type compared to an edgetic non-pathogenic mutation in the presence of false negatives, which results in a higher probability for quasi-wild-type mutations to be mildly deleterious and a lower probability for edgetic mutations to be mildly deleterious. Therefore, our estimated probability for quasi-wild-type mutations to be effectively neutral (>∼40%) and our estimated probability for edgetic mutations to be mildly deleterious (>∼75%) both represent lower bounds in the presence of false negatives in PPI datasets.

While our estimates of mutation fitness effect are robust to experimental noise, our datasets that were used for structure-based calculations still contain other biases. First, literature-curated PPIs in Lit-SI are enriched for interactions of functional and disease importance, a bias that may affect our estimates of fitness effect for edgetic mutations. We address this bias by additionally examining systematically mapped PPIs in Y2H-SI. Second, experimentally determined 3D structures in PDB are also biased towards interactions with functional and disease importance. We partially address this bias by using homology models in addition to experimental 3D structures of proteins and PPIs. Furthermore, we complement our mutation edgotype predictions with mutation edgotypes determined by experiments. These experimentally determined mutation edgotypes are free from the aforementioned biases and approximations that are present in our predictions. The broad agreement between computation and experiment indicates that our estimates are robust against these biases and approximations.

Our structure-based calculations also include several numerical approximations. First, we define a mutation to be exposed on protein surface if its RSA is larger than 0.25. Although this cut-off may vary slightly between different structural biology studies, we chose the cut-off that was found to best segregate the interior residues of a protein from the exterior residues (Levy, 2010). Second, we define a residue to be at the binding interface of a protein if it falls within a distance of 5Å from any residue in the interaction partner. This distance cut-off for defining interface residues has been found to be the optimal cut-off for detecting residue contacts within protein structures (Salamanca Viloria et al., 2017), and is widely used in structural biology studies (Dai et al., 2016; Espadaler et al., 2005; Ghadie and Xia, 2019; Nadalin and Carbone, 2018; Winter et al., 2006; Yang et al., 2019). Other studies have used slightly different cut-offs such as 6 Å (Cukuroglu et al., 2014; Davis and Sali, 2005; Ofran and Rost, 2003). To see whether our results are robust to different choices of cut-offs, we repeated our mutation edgotype predictions in both interactomes Y2H-SI and Lit-SI using two other distance cut-offs for defining residues at the binding interface, 4 and 6 Å. We then re-calculated the fitness effects for mutation edgotypes based on these new cut-offs. Our estimates of fitness effect remain unchanged (Supplementary Figures S2,S3), thus proving that our edgotype predictions and fitness effect calculations are robust to different choices of distance cut-off for defining PPI interface residues.

Furthermore, we predict an interfacial mutation to be edgetic if it causes a change in PPI binding free energy ∆∆G > 0.5 kcal/mol. This ∆∆G cut-off for edgetic mutations has been previously established and used by other structural biology studies (Cui et al., 2019). While using a different ∆∆G cut-off may change the proportion of edgetic mutations among both neutral and deleterious mutations, our estimates of fitness effect are robust to small variations in our choice of binding ∆∆G cut-off. For quasi-null mutations in particular, by setting the edgotype variable T in Eqs 2–4 in the Methods section to quasi-null (QN), it is clear that the fitness effect probabilities for quasi-null mutations *P* (N|QN), *P* (M|QN) and *P* (S|QN) depend only on the proportion of quasi-null mutations among neutral (N), mildly deleterious (M) and strongly detrimental (S) mutations: *P* (QN|N), *P* (QN|M) and *P* (QN|S), and do not depend on the proportions of edgetic or quasi-wild-type mutations. Since interfacial mutations are typically not buried and therefore cannot be predicted by our method to be quasi-null, a change in our binding ∆∆G cut-off can only change the edgotype of an interfacial mutation from edgetic to quasi-wild-type or vice versa, thus leaving the proportion of quasi-null mutations among both neutral and deleterious mutations and their fitness effect estimates unchanged.

At the same time, by setting the edgotype variable T in Eqs 5–7 in the Methods section to either edgetic or quasi-wild-type, it is easy to see that the fitness effect probabilities for edgetic (E) mutations and quasi-wild-type (QW) mutations to be neutral (N), mildly deleterious (M) or strongly detrimental (S): *P* (N|T), *P* (M|T) and *P* (S|T) where T = E or QW, depend only on the ratio of proportions *P* (E|N)/*P* (E|M) for edgetic mutations and *P* (QW|N)/*P* (QW|M) for quasi-wild-type mutations (after substituting *P* (T|S) = 0 under Assumption I and *P* (T|S) = *P* (T|M) under Assumption II). To see whether these two ratios of proportions change for different binding ∆∆G cut-offs, we repeated our edgotype predictions using three different cut-offs: 0.3, 0.5 and 0.7 kcal/mol, and re-calculated the ratios *P* (E|N)/*P* (E|M) and *P* (QW|N)/*P* (QW|M) for each cut-off. Our results show that these two ratios remain almost unchanged for the three ∆∆G cut-offs (Supplementary Table S3), indicating that our estimates of fitness effect for edgetic mutations and quasi-wild-type mutations are also robust to different choices of binding ∆∆G cut-off used for predicting edgetic mutations.

Our edgotype predictions make use of the change in free energy (∆∆G) upon mutation as predicted by the widely known method FoldX (Schymkowitz et al., 2005). Other computational methods are also available for predicting ∆∆G upon mutation, including mCSM-PPI2 (Rodrigues et al., 2019) for PPI binding free energy and DynaMut2 (Rodrigues et al., 2020) for protein folding free energy. Unlike FoldX which predicts ∆∆G values using only physics-based calculations, the other aforementioned methods make use of protein sequence and evolutionary information which may introduce biases into our edgotype predictions for neutral and deleterious mutations. Furthermore, these methods do not offer the option of predicting ∆∆G values for thousands of mutations simultaneously, which is necessary for our large-scale study. Nonetheless, to check whether our fitness effect estimates are robust to different choices of ∆∆G prediction methods, we repeated our mutation edgotype predictions on a sample of mutations in both interactomes Y2H-SI and Lit-SI (137 mutations in Y2H-SI, and 202 mutations in Lit-SI), this time using mCSM-PPI2 for predicting change in PPI binding free energy and DynaMut2 for predicting change in protein folding free energy. We repeated our fitness effect calculations using these new edgotype predictions and our estimates of fitness effect remain broadly consistent with our FoldX-based estimates (Figure S4), thus proving that our fitness effect calculations are robust to different choices of methods for predicting ∆∆G upon mutation.

In addition, we further validated our edgotype prediction method using FoldX-based ∆∆G calculations on the experimental dataset of Sahni et al. (2015). For predicting edgetic mutations, we obtained a true positive rate (TPR) of 0.3 and a false positive rate (FPR) of 0.04 (*p* = 0.002, two-sided Fisher’s exact test). For predicting quasi-null mutations, we obtained a TPR of 0.56 and a FPR of 0.07 (*p* = 0.001, two-sided Fisher’s exact test). A TPR that is equal to FPR indicates that predictions are not better than random expectation. Our TPR for predicting edgetic mutations is 7.5 times larger than the FPR, and our TPR for predicting quasi-null mutations is 8 times larger than the FPR, proving that our structure-based method for predicting mutation edgotype is of very high quality.

Our structure-based calculations make a clear distinction between quasi-null mutations and edgetic mutations. We consider a mutation to be edgetic if and only if it disrupts at least one PPI by disrupting the binding interface, and we consider a mutation to be quasi-null if it disrupts all interactions by disrupting overall protein stability. In the experimental dataset of Sahni et al., the definition of quasi-null mutations is less straightforward. There, due to the lack of structural information, a mutation is considered to be edgetic if it disrupts some but not all interactions, and a mutation is considered to be quasi-null if it disrupts all interactions. It is possible for an edgetic mutation to disrupt all interactions without disrupting overall protein stability if all interactions are mediated by the same interface. In that case, an edgetic mutation in the dataset of Sahni et al. will be misclassified as quasi-null. We address this experimental caveat by performing structure-based computations of mutation edgotypes, which are free from this caveat as explained above. The broad agreement between computation and experiment shows that our estimates obtained from experiments are robust to such potential errors.

Deleterious mutations that have different edgotypes may also have different physiochemical properties. Based on amino acid biochemical properties provided by National Center for Biotechnology Information (NCBI) (1988), we found that 61% of deleterious quasi-null mutations identified by both predictions and experiments involve a decrease in residue hydrophobicity upon mutation, compared to only 49% for edgetic mutations and 44% for quasi-wild-type mutations (Supplementary Figure S5A), consistent with the expectation that buried quasi-null mutations disrupt overall protein stability. We also found that 67% of deleterious quasi-null mutations involve an increase in residue molecular weight upon mutation, compared to only 43% for edgetic mutations and 48% for quasi-wild-type mutations (Supplementary Figure S5B), also consistent with the expectation that buried quasi-null mutations disrupt overall protein stability. Since strongly detrimental mutations are expected to be predominantly quasi-null rather than edgetic or quasi-wild-type, the distinct physiochemical patterns of (mildly) deleterious mutations that are quasi-null suggest that strongly detrimental mutations are also more likely to involve a decrease in residue hydrophobicity and an increase in residue molecular weight upon mutation compared to the average deleterious mutation.

In theory, quasi-null mutations are likely to cause complete loss of protein function, similar to gene knockout. Using the Achilles dataset of CRISPR gene knockout effects in 808 cancer cell lines provided by the DepMap project (Dempster et al., 2019; DepMap, 2020; Meyers et al., 2017), we quantified the knockout effect for all genes that encode proteins disrupted by quasi-null mutations in both predictions and experiments. We found that genes corresponding to proteins disrupted by deleterious quasi-null mutations have a more detrimental knockout effect on average across all cell lines compared to genes corresponding to proteins that are disrupted by non-deleterious quasi-null mutations (*p* < 10^−29^ in all interactomes, two-sided *t*-test; Supplementary Figure S5C). These positive correlations in fitness effect between protein disruption by quasi-null mutations and corresponding gene knockout suggest that deleterious mutations tend to disrupt proteins of higher functional importance compared to neutral mutations.

Proteins encoded by essential genes often show distinct network properties. To examine whether mutation edgotypes among essential genes show patterns of fitness effect that are distinct from other genes in the interactome, we repeated our calculations of edgotype fitness effect this time by predicting mutation edgotypes based on whether or not they disrupt PPIs of essential genes only. Here, we maintain our original assumption that strongly detrimental mutations are predominantly quasi-null, with the probability of disrupting proteins of essential genes equal to the overall fraction of essential genes in the interactome. Overall, we observed a slight increase in the probability for quasi-wild-type mutations among essential genes to be mildly deleterious compared to the average gene in the interactome (Supplementary Figure S6), which is expected since mutations that do not disrupt PPIs of essential genes may still disrupt PPIs of other genes. At the same time, we observed a slight increase in the probability for edgetic mutations among essential genes to be mildly deleterious compared to the average gene in the interactome (Supplementary Figure S6), suggesting that PPIs of essential genes may be more important to cellular function than PPIs of other genes. On the other hand, we observed a significant increase in the probability for quasi-null mutations among essential genes to be strongly detrimental, with its upper limit reaching ∼100% compared to ∼75% for the average gene in the interactome (Supplementary Figure S6). This significant increase reflects the essentiality of proteins encoded by essential genes compared to proteins of other genes.

Our results reveal that while common mutations rarely disrupt the interactome, pathogenic mutations are significantly more likely to disrupt the interactome, either disrupting specific PPIs by disrupting the binding interface (edgetic) or disrupting all PPIs by disrupting overall protein stability (quasi-null), thus leading to loss of function in both cases. On the other hand, while quasi-wild-type mutations do not disrupt pre-existing PPIs, it is possible for some pathogenic quasi-wild-type mutations to create new PPIs by creating new binding interfaces, thus leading to gain of function (Yates and Sternberg, 2013). Gain-of-function mutations are known to be associated with different disease phenotypes (Li and Babu, 2018; Meyer et al., 2018), including cancers (Kakiuchi et al., 2014; van Oijen and Slootweg, 2000) and neurodegenerative diseases (Lashuel et al., 1999). While such mutations are hard to detect by systematic experiments or computational predictions, recent genome-wide screens suggest that gain-of-interaction mutations are ∼30 times less likely to occur in human disease than edgetic loss-of-interaction mutations (Sahni et al., 2015). Nonetheless, our estimates of fitness effect for different mutation edgotypes are independent of the extent of gain-of-function mutations in the interactome. Our definitions for mutation edgotypes refer only to mutations that either disrupt pre-existing PPIs in the reference interactome (edgetic or quasi-null) or do not disrupt any pre-existing PPIs (quasi-wild-type), and are independent of the extent of gain-of-function mutations. Moreover, the three prior probabilities in our Bayesian framework *P*(N), *P*(M) and *P*(S), for new missense mutations to be neutral (N), mildly deleterious (M) and strongly detrimental (S) are obtained from population genetics studies using procedures that are robust to gain-of-function mutations (Kryukov et al., 2007). While gain-of-function mutations are beyond the scope of our current study and do not affect our estimates of fitness effect for different mutation edgotypes among pre-existing PPIs, our Bayesian framework can be extended in the future to the calculation of fitness effect for quasi-wild-type mutations that specifically cause gain of function. In that case, a more granular definition of fitness effect such as “likely neutral” and “likely deleterious” may be helpful when such phenotype data becomes available. Finally, our Bayesian framework can also be extended to the calculation of fitness effect for co-occurring mutations (Skoulidis and Heymach, 2019) when enough data becomes available.

In summary, we estimate that at least ∼40% of mutations that do not disrupt the interactome (quasi-wild-type mutations) are effectively neutral, and that the remaining are mostly mildly deleterious rather than strongly detrimental. These results suggest that some mutations that do not disrupt PPIs may cause disease by disrupting alternative molecular interactions such as protein-DNA interactions (Fuxman Bass et al., 2015; Reece-Hoyes et al., 2011; Sahni et al., 2015) and protein-chemical interactions (Reva et al., 2011; Sahni, et al., 2015), or by the event of creating new interactions (Li and Babu, 2018; Meyer et al., 2018). We also estimate that the vast majority (>∼75%) of edgetic mutations are mildly deleterious rather than strongly detrimental, consistent with expectations from previous studies (Mosca et al., 2015; Sahni et al., 2015; Wang et al., 2012) and also suggesting that the majority of human PPIs are under strong purifying selection. Finally, we estimate that the vast majority (>∼95%) of quasi-null mutations are either mildly deleterious or strongly detrimental, with as low as ∼25% being mildly deleterious and up to ∼75% being strongly detrimental, indicating that disrupting overall protein stability is much more likely to be strongly detrimental to the cell than disrupting a single PPI, and also suggesting that the stability of most human proteins is essential to human life. These estimates represent a genome-wide average over the entire human interactome, likely with significant variations within the interactome. Indeed, certain subsets of the interactome appear to be more dispensable than others (Landry et al., 2009; Studer et al., 2016). Our study further demonstrates the important role of systematic mapping of interactome perturbation patterns in elucidating the phenotypic consequences of genetic mutations, and the power of complementing experimental studies of interactome perturbations with high-resolution structural biology computations.

## Methods

### Constructing Protein-Protein Interaction Structural Models

Protein complex structures at atomic resolution were obtained from the Protein Data Bank (PDB) (Berman et al., 2003). For structures containing more than one model, the first model was selected. Gene Ensembl IDs in the HuRI reference interactome were mapped to protein UniProt IDs and corresponding amino acid sequences using the ID mapping table provided by UniProt (The UniProt Consortium, 2014). For proteins in the IntAct reference interactome, UniProt IDs provided by the IntAct database were used to obtain corresponding amino acid sequences. Next, we used BLAST (Altschul et al., 1990) to perform sequence alignment of all protein sequences against all PDB chain sequences found in PDB’s SEQRES records, with an E-value cut-off of 10^−5^. For each pair of protein sequence and PDB chain, the alignment with the smallest E-value was retained, and the remaining alignments were discarded. A PPI was annotated with a pair of chains found in the same PDB structure if: 1) the two chains had a binding interface, 2) one of the proteins in the PPI has a sequence alignment with one of the chains in the chain pair, with ≥50% of interface residues mapped onto the protein; and 3) the other protein in the PPI has a sequence alignment with the other chain in the chain pair, with ≥50% of interface residues mapped onto the protein. PPIs having no PDB chain-pair annotations were discarded. The 3D structure corresponding to the annotated chain-pair of each PPI was selected as a template for generating the PPI structural model. We then used BLAST again to generate the sequence alignment for each PPI against the residues that have 3D coordinates in the template structure file. Finally, we used the MODELLER library (version 9.23) (Webb and Sali, 2016) to construct a structural model for each PPI starting from its template structure.

### Defining Binding Interfaces in Protein-Protein Interaction Structural Models

We calculated the pairwise Euclidean distance between all residues of the first protein and all residues of the second protein. The distance between two residues was calculated as the minimum distance between all atoms of the first residue and all atoms of the second residue. If the residue of one protein is within a distance of 5 Å from any residue in the other protein, that residue was labelled as an interface residue.

### Mapping Pathogenic Mutations Onto the Human Structural Interactome

Germline mutations in human with associated phenotypic consequences were retrieved in February 2020 from the ClinVar database (genome assembly GRCh38) (Landrum et al., 2016). We selected missense mutations that are strictly labelled as pathogenic only, with supporting evidence (i.e., with at least one star), and with no conflicting phenotypic interpretations. To map mutations onto proteins in the human structural interactome, we searched the protein’s RefSeq transcript provided by ClinVar for the mutation flanking sequence, defined as either the first 10 amino acid residues or all amino acid residues, whichever one is shorter, on both sides of the mutation. Then we searched the protein’s sequence designated by UniProt for the mutation flanking sequence obtained from the RefSeq transcript. If the flanking sequence was found on the protein sequence at the same position reported by ClinVar, the mutation was retained for further analysis, otherwise the mutation was discarded. For multiple mutations mapping onto the same position, only one mutation was retained for further analysis.

### Mapping Common Mutations Onto the Human Structural Interactome

Single Nucleotide Polymorphism (SNP) mutations in human were retrieved in February 2020 from the Single Nucleotide Polymorphism Database (dbSNP) (build 150 GRCh38p7) (Sherry et al., 2001). First, we selected only missense SNPs that are labelled as validated and not withdrawn, and are assigned a location on the RefSeq transcript of a protein. Next, we discarded all mutations labelled with pathogenic or uncertain assertions (e.g., pathogenic, likely pathogenic, drug-response, uncertain significance or other). Then we selected mutations that have minor allele frequencies ≥1%, as common mutations with high frequencies are unlikely to be associated with any disease. To map mutations onto proteins in the human structural interactome, we searched the protein’s RefSeq transcript provided by dbSNP for the mutation flanking sequence, defined as either the first 10 amino acid residues or all amino acid residues, whichever one is shorter, on both sides of the mutation. Then we searched the protein’s sequence designated by UniProt for the mutation flanking sequence obtained from the RefSeq transcript. If the flanking sequence was found on the protein sequence at the same position reported by dbSNP, the mutation was retained for further analysis, otherwise the mutation was discarded. Finally, mutations overlapping in position with pathogenic mutations were also discarded.

### Calculating Residue Relative Solvent Accessibility

The absolute solvent accessibility (ASA) of the residue was calculated using Biopython’s DSSP module. The residue’s relative solvent accessibility (RSA) was calculated by dividing the residue’s ASA by the 99.99th percentile of its corresponding amino acid ASA distribution among all PDB structures, as provided in DSSP’s pre-calculated ASA file.

### Calculating Edgotype Fitness Effect

The edgotype of a mutation can be either edgetic, quasi-null, or quasi-wild-type. In addition, the fitness effect of a mutation can be either neutral, mildly deleterious, or strongly detrimental. Given a set of neutral and mildly deleterious mutations with known edgotypes, we calculate the fitness effect for mutations of specific edgotype *T* using the following procedure: From the mutation edgotype data, we obtain the probabilities for effectively neutral (N), mildly deleterious (M), and strongly detrimental (S) mutations to be of edgotype *T*: *P* (T|N), *P* (T|M), and *P* (T|S), where *P* (T|S) = {1 if *T* is quasi-null, and 0 if *T* is quasi-wild-type or edgetic} assuming that strongly detrimental mutations are quasi-null rather than edgetic or quasi-wild-type. Next, we obtain from (Kryukov et al., 2007) the probabilities for new missense mutations to be effectively neutral (N), mildly deleterious (M), or strongly detrimental (S): *P* (N) = 27%, *P* (M) = 53%, *P* (S) = 20%. We then integrate these numbers to calculate the probability for a new missense mutation to be of edgotype *T*:

P(T)=P(T|N)P(N)+P(T|M)P(M)+P(T|S)P(S) (1)

Finally, we apply Bayes’ theorem *P* (A|B) = *P* (B|A)*P* (A)/*P* (B) to calculate the probability for a mutation of edgotype *T* to be effectively neutral (N), mildly deleterious (M) or strongly detrimental (S):

$$P\left(N\text{|}T\right)=\frac{P\left(T|N\right)P\left(N\right)}{P\left(T\right)}$$ (2)

$$P\left(M\text{|}T\right)=\frac{P\left(T|M\right)P\left(M\right)}{P\left(T\right)}$$ (3)

$$P\left(S\text{|}T\right)=\frac{P\left(T|S\right)P\left(S\right)}{P\left(T\right)}$$ (4)

Now, we describe procedures for calculating the 95% confidence intervals for these three edgotype fitness effect probabilities. By substituting the value of *P* (T) from Eq. 1 into Eq. 2, *P* (N|T) can be written as follows:

$$\frac{1}{P\left(N\text{|}T\right)}=1+\frac{P\left(T\text{|}M\right)\frac{P\left(M\right)}{P\left(N\right)}+\frac{P\left(T\text{|}S\right)P\left(S\right)}{P\left(N\right)}}{P\left(T\text{|}N\right)}=1+\frac{a\times P\left(T\text{|}M\right)+b}{P\left(T\text{|}N\right)}$$ (5)

where $a=\frac{P\left(M\right)}{P\left(N\right)}$ and. $b=\frac{P\left(T|S\right)P\left(S\right)}{P\left(N\right)}$

The 95% confidence interval for the ratio {a × *P* (T|M) + *b*}/*P* (T|N) was calculated according to Bland (Bland, 2015), which was then used to calculate the 95% confidence interval for *P* (N|T) using the above equation.

Similarly, by substituting the value of *P* (T) from Eq. 1 into Eq. 3, *P* (M|T) can be written as follows:

$$\frac{1}{P\left(M\text{|}T\right)}=1+\frac{P\left(T\text{|}N\right)\frac{P\left(N\right)}{P\left(M\right)}+\frac{P\left(T\text{|}S\right)P\left(S\right)}{P\left(M\right)}}{P\left(T\text{|}M\right)}=1+\frac{a\times P\left(T\text{|}N\right)+b}{P\left(T\text{|}M\right)}$$ (6)

where $a=\frac{P\left(N\right)}{P\left(M\right)}$ and. $b=\frac{P\left(T|S\right)P\left(S\right)}{P\left(M\right)}$

The 95% confidence interval for the ratio {a×P(T|N)+b}/P(T|M) was calculated according to Bland (Bland, 2015), which was then used to calculate the 95% confidence interval for *P* (M|T) using the above equation.

Finally, by substituting the value of *P* (T) from Eq. 1 into Eq. 4, *P* (S|T) can be written as follows:

$$\frac{1}{P\left(S\text{|}T\right)}=1+\frac{P\left(T\text{|}N\right)P\left(N\right)+P\left(T\text{|}M\right)P\left(M\right)}{P\left(T\text{|}S\right)P\left(S\right)}=1+\left\{a\times P\left(T\text{|}N\right)\right\}+\left\{b\times P\left(T\text{|}M\right)\right\}$$ (7)

where $a=\frac{P\left(N\right)}{P\left(T|S\right)P\left(S\right)}$ and. $b=\frac{P\left(M\right)}{P\left(T|S\right)P\left(S\right)}$

The 95% confidence interval for the sum {a×P(T|N)}+{b×P(T|M)} was calculated according to Bland (Bland, 2015), which was then used to calculate the 95% confidence interval for *P* (S|T) using the above equation.

## Data Availability

The original contribution presented in the study are included in the article/Supplementary Material, further inquiries can be directed to the corresponding author.
