# Supplementary material for: Mutation Edgotype Drives Fitness Effect in Human
Source: Front Bioinform. 2021 Aug 30;1:690769. doi: 10.3389/fbinf.2021.690769 (PMC9581054; doi:10.3389/fbinf.2021.690769)
Supplement: Supplementary file 3 [file DataSheet1.PDF]

Mutation edgotype drives fitness effect in human

Supplementary Information

Ghadie and Xia

**a**	Quasi-wild-type			Edgetic			Quasi-null		
	**N** (%)	**M** (%)	**S** (%)	**N** (%)	**M** (%)	**S** (%)	**N** (%)	**M** (%)	**S** (%)
Y2H-SI	40	60	0	6	94	0	2	25	73
Lit-SI	41	59	0	11	89	0	3	36	61
Experiment	52	48	0	6	94	0	3	39	58

**b**	Quasi-wild-type			Edgetic			Quasi-null		
	**N** (%)	**M** (%)	**S** (%)	**N** (%)	**M** (%)	**S** (%)	**N** (%)	**M** (%)	**S** (%)
Y2H-SI	38-42	58-62	NA	3-10	90-97	NA	1-3	20-31	67-78
Lit-SI	40-42	58-60	NA	8-14	86-92	NA	2-4	33-39	59-64
Experiment	48-57	43-52	NA	2-21	79-98	NA	1-12	30-49	51-63

Table S1 Fitness effect for different mutation edgotypes in human obtained under the assumption that strongly detrimental mutations are all quasi-null
(a) Probabilities for quasi-wild-type mutations, edgetic mutations, and quasi-null mutations to be effectively neutral (N), mildly deleterious (M), or strongly detrimental (S) in human, assuming that strongly detrimental mutations are all quasi-null (Assumption I). Probabilities were estimated from mutation edgotypes obtained by structure-based calculations in the two human structural interactomes, Y2H-SI and Lit-SI, and from mutation edgotypes obtained by experiments. **(b)** 95% confidence intervals for the fitness effect probabilities presented in (a).

**a**	Quasi-wild-type			Edgetic			Quasi-null		
	**N** (%)	**M** (%)	**S** (%)	**N** (%)	**M** (%)	**S** (%)	**N** (%)	**M** (%)	**S** (%)
Y2H-SI	33	49	18	4	70	26	5	69	26
Lit-SI	34	48	18	8	67	25	5	69	26
Experiment	44	41	15	5	69	26	6	68	26

**b**	Quasi-wild-type			Edgetic			Quasi-null		
	**N** (%)	**M** (%)	**S** (%)	**N** (%)	**M** (%)	**S** (%)	**N** (%)	**M** (%)	**S** (%)
Y2H-SI	31-34	47-51	18-19	2-7	62-76	21-32	3-7	62-76	21-32
Lit-SI	33-34	47-49	18-19	6-11	62-72	22-28	4-6	66-72	24-28
Experiment	40-48	36-45	14-17	1-16	56-80	22-30	2-19	54-80	21-31

Table S2 Fitness effect for different mutation edgotypes in human obtained under the assumption that strongly detrimental mutations are similar to mildly deleterious mutations
(a) Probabilities for quasi-wild-type mutations, edgetic mutations, and quasi-null mutations to be effectively neutral (N), mildly deleterious (M), or strongly detrimental (S) in human, assuming that strongly detrimental mutations are as likely as mildly deleterious mutations to be quasi-wild-type, edgetic or quasi-null (Assumption II). Probabilities were estimated from mutation edgotypes obtained by structure-based calculations in the two human structural interactomes, Y2H-SI and Lit-SI, and from mutation edgotypes obtained by experiments. **(b)** 95% confidence intervals for the fitness effect probabilities presented in (a).

	ΔΔG cut-off	P(E|N)	P(E|M)	$\frac{P(E\|N)}{P(E\|M)}$	P(QW|N)	P(QW|M)	$\frac{P(QW\|N)}{P(QW\|M)}$	P(QN|N)	P(QN|M)	$\frac{P(QN\|N)}{P(QN\|M)}$
Y2H-SI	0.3	2%	15.7%	**0.13**	96.4%	71.7%	**1.34**	1.7%	12.6%	**0.13**
	0.5	1.5%	12.6%	**0.12**	96.8%	74.2%	**1.30**	1.7%	13.2%	**0.13**
	0.7	1.3%	11.3%	**0.12**	97%	75.5%	**1.28**	1.7%	13.2%	**0.13**
Lit-SI	0.3	2.8%	10.6%	**0.26**	93.9%	67.6%	**1.39**	3.3%	21.8%	**0.15**
	0.5	2%	8.5%	**0.24**	94.7%	69.4%	**1.36**	3.3%	22.1%	**0.15**
	0.7	1.5%	7.7%	**0.20**	95.2%	70%	**1.36**	3.3%	22.3%	**0.15**

Table S3 Proportions of mutation edgotype derived from different binding ΔΔG cut-offs
Probabilities for neutral (N) and mildly deleterious (M) mutations to be edgetic (E), quasi-wild-type (QW) or quasi-null (QN), derived from different PPI binding ΔΔG cut-offs used for predicting edgetic mutations in the two human structural interactomes, Y2H-SI and Lit-SI.

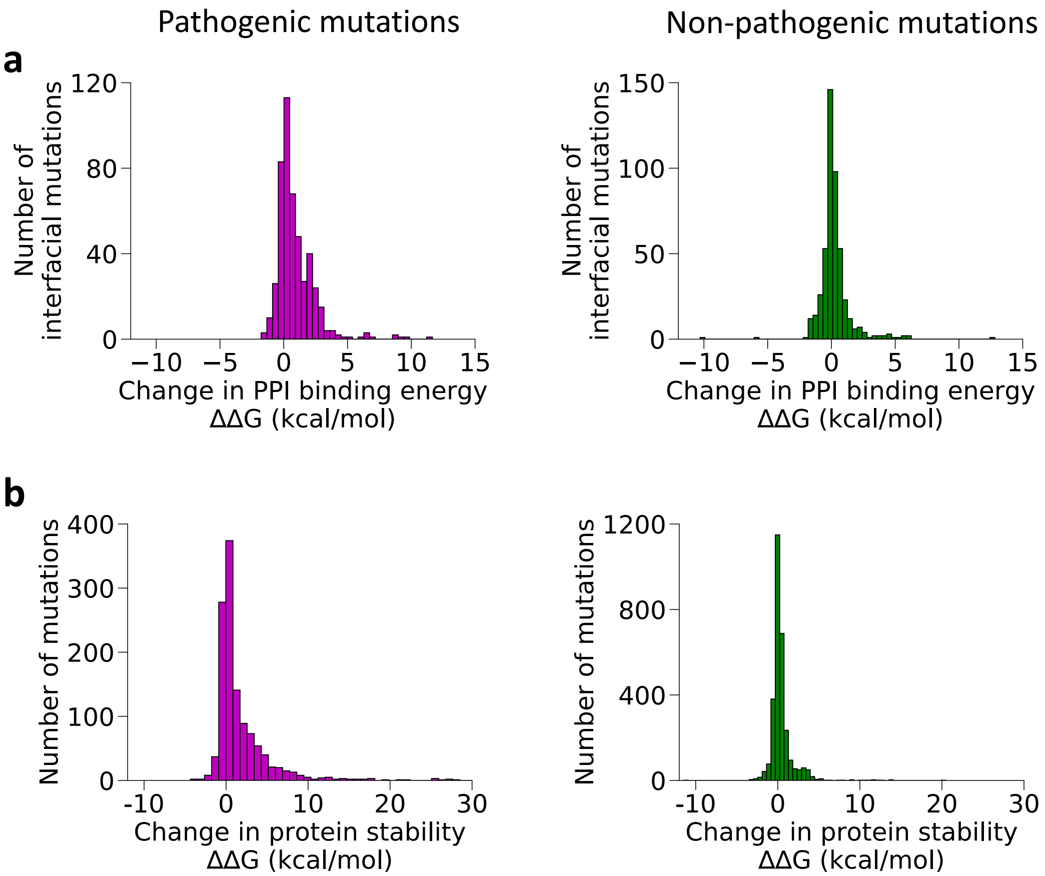

Figure S1 Change in protein binding and folding free energy upon mutation
(a) Change in PPI binding free energy (ΔΔG) distribution for all pathogenic and non-pathogenic interfacial mutations in both structural interactomes Y2H-SI and Lit-SI. **(b)** Change in protein folding free energy (ΔΔG) distribution for all pathogenic and non-pathogenic mutations in both structural interactomes Y2H-SI and Lit-SI. ΔΔG values were calculated using FoldX.

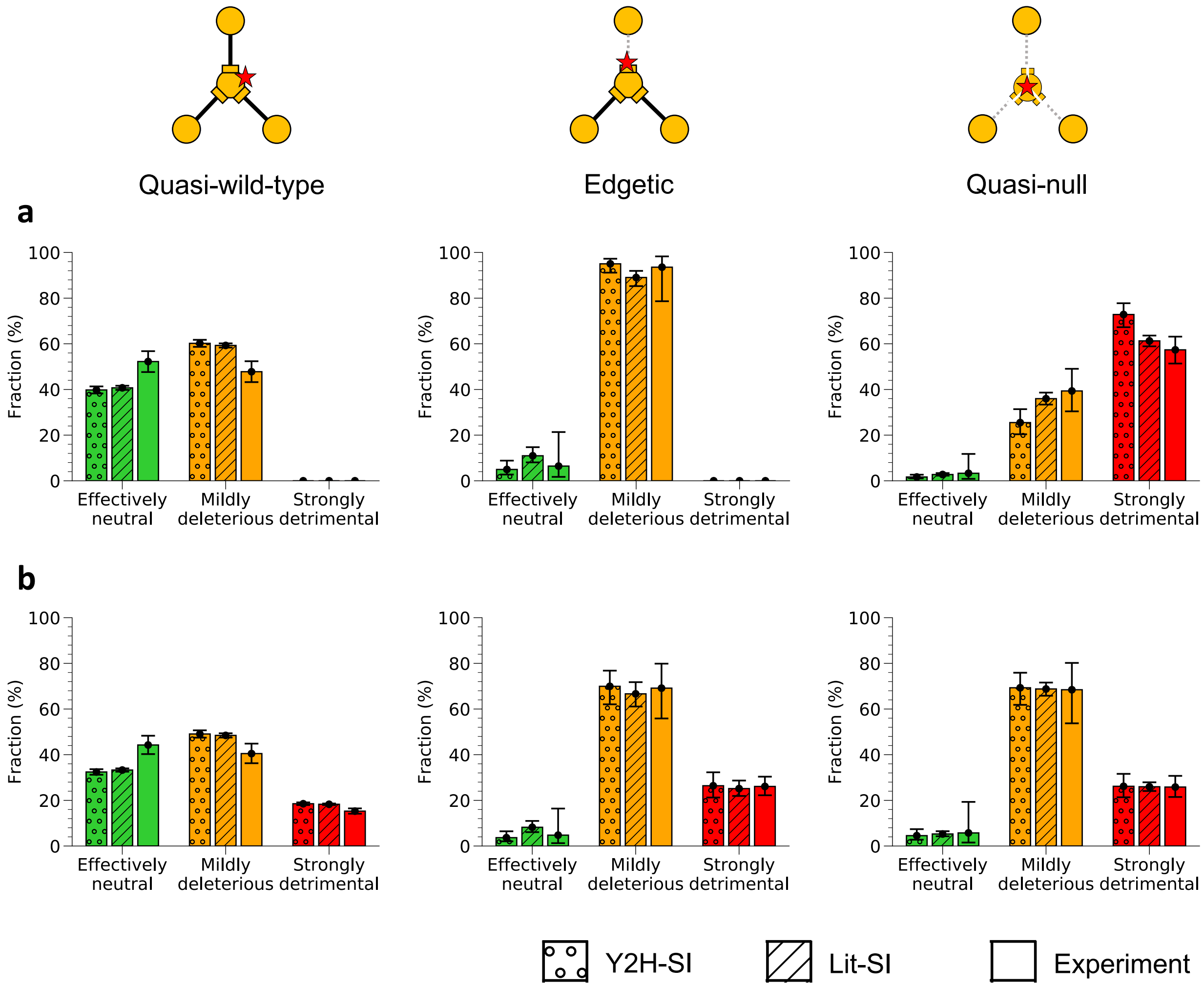

Figure S2 Edgotype fitness effect calculated with PPI interface distance ≤ 4Å
Probabilities for quasi-wild-type mutations (left), edgetic mutations (middle), and quasi-null mutations (right) to be effectively neutral, mildly deleterious, or strongly detrimental in human. Only residues at a distance ≤ 4Å from the interaction partner were considered to be interfacial. Probabilities were estimated from mutation edgotypes obtained by structure-based predictions in the two human structural interactomes, Y2H-SI and Lit-SI, and from mutation edgotypes obtained by experiments. **(a)** Fitness effect calculated assuming that strongly detrimental mutations are all quasi-null (Assumption I). **(b)** Fitness effect calculated assuming that strongly detrimental mutations are as likely as mildly deleterious mutations to be quasi-wild-type, edgetic or quasi-null (Assumption II). Error bars represent 95% confidence intervals.

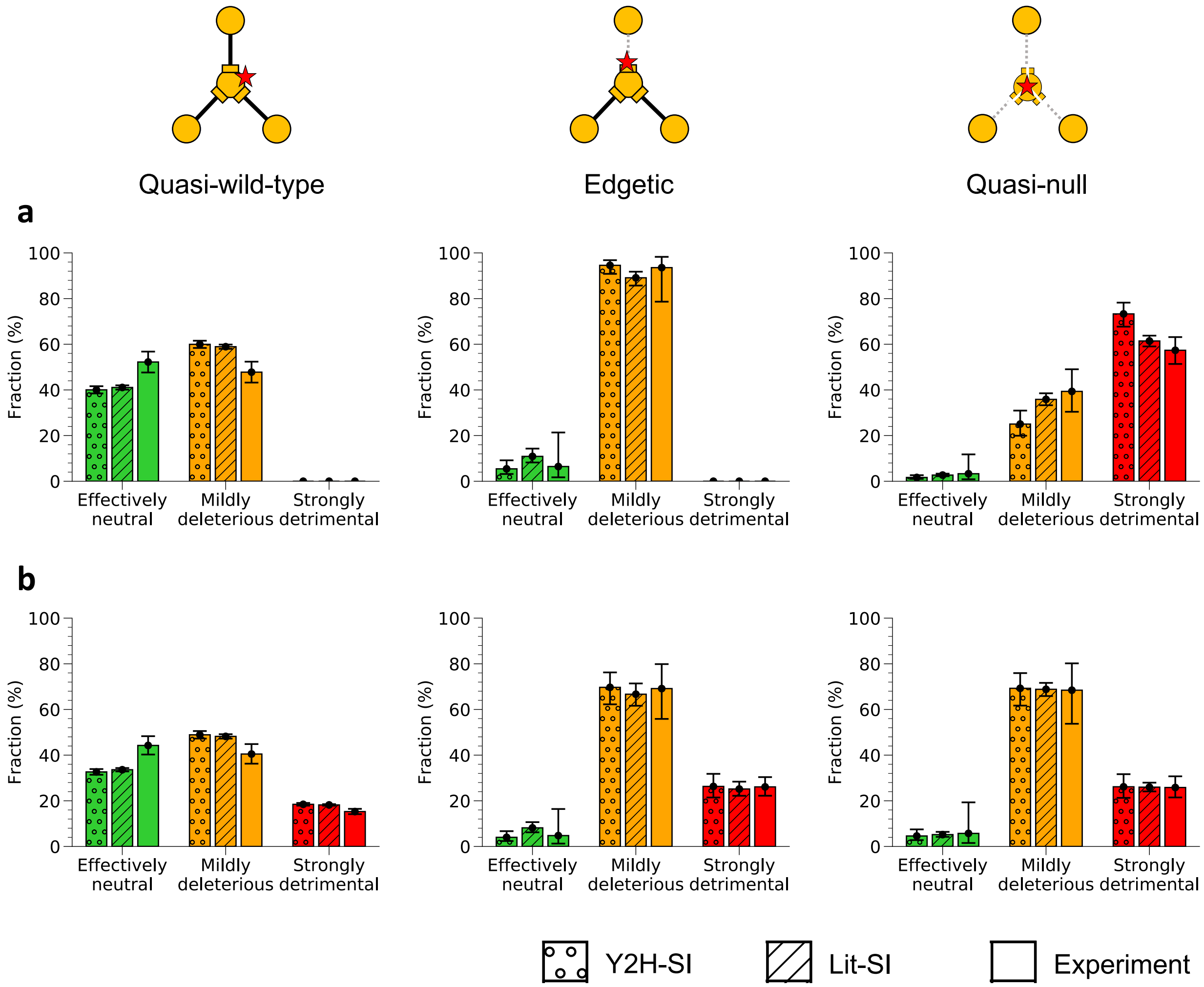

Figure S3 Edgotype fitness effect calculated with PPI interface distance ≤ 6Å
Probabilities for quasi-wild-type mutations (left), edgetic mutations (middle), and quasi-null mutations (right) to be effectively neutral, mildly deleterious, or strongly detrimental in human. Only residues at a distance ≤ 6Å from the interaction partner were considered to be interfacial. Probabilities were estimated from mutation edgotypes obtained by structure-based predictions in the two human structural interactomes, Y2H-SI and Lit-SI, and from mutation edgotypes obtained by experiments. **(a)** Fitness effect calculated assuming that strongly detrimental mutations are all quasi-null (Assumption I). **(b)** Fitness effect calculated assuming that strongly detrimental mutations are as likely as mildly deleterious mutations to be quasi-wild-type, edgetic or quasi-null (Assumption II). Error bars represent 95% confidence intervals.

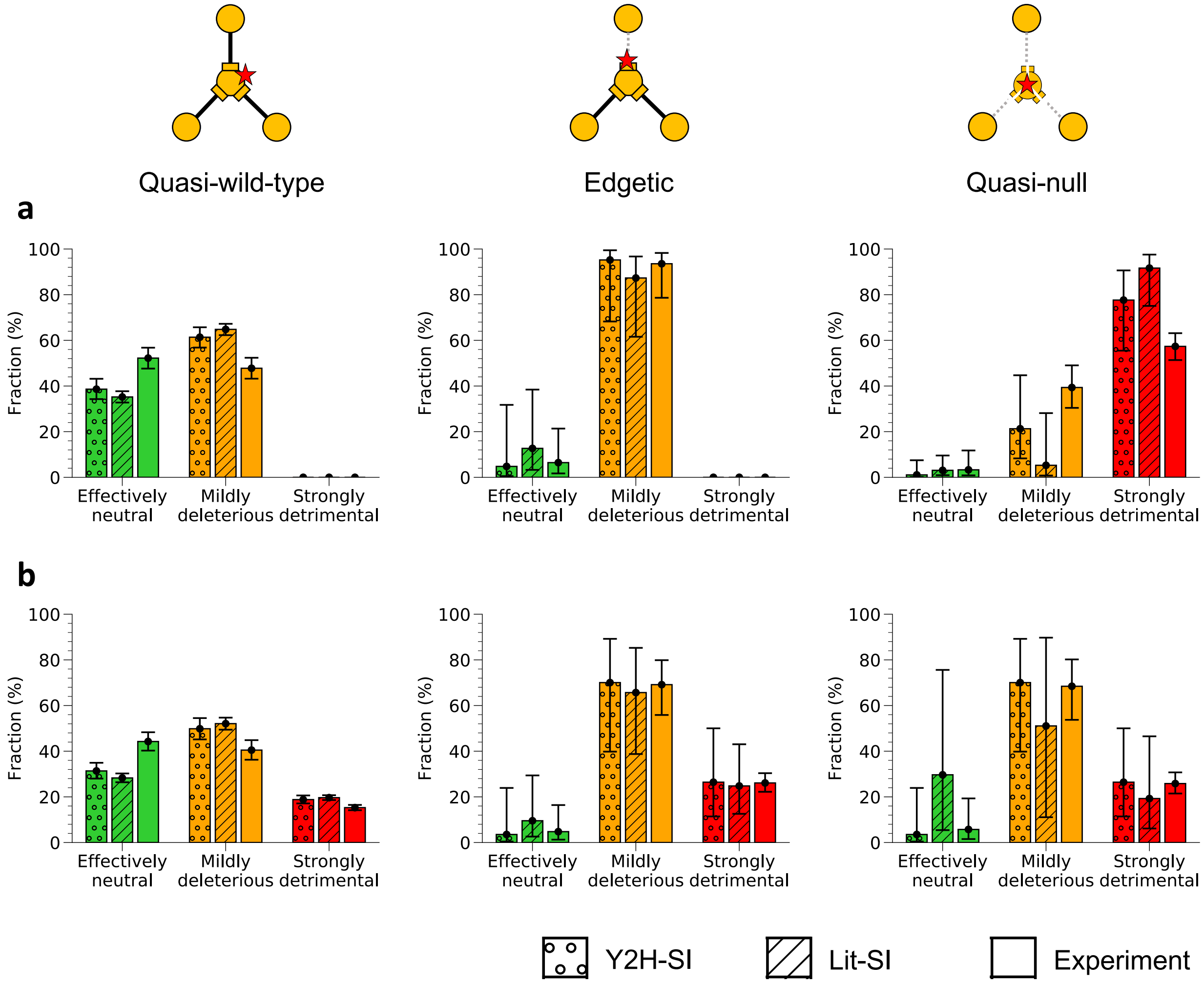

Figure S4 Edgotype fitness effect derived from ΔΔG values calculated by mCSM-PPI2 and DynaMut2

Probabilities for quasi-wild-type mutations (left), edgetic mutations (middle), and quasi-null mutations (right) to be effectively neutral, mildly deleterious, or strongly detrimental in human. Change in binding free energy upon mutation for interfacial residues was calculated using mCSM-PPI2. Change in folding free energy upon mutation for buried residues was calculated using DynaMut2. Probabilities were estimated from mutation edgotypes obtained by structure-based predictions in the two human structural interactomes, Y2H-SI and Lit-SI, and from mutation edgotypes obtained by experiments. **(a)** Fitness effect calculated assuming that strongly detrimental mutations are all quasi-null (Assumption I). **(b)** Fitness effect calculated assuming that strongly detrimental mutations are as likely as mildly deleterious mutations to be quasi-wild-type, edgetic or quasi-null (Assumption II). Error bars represent 95% confidence intervals.

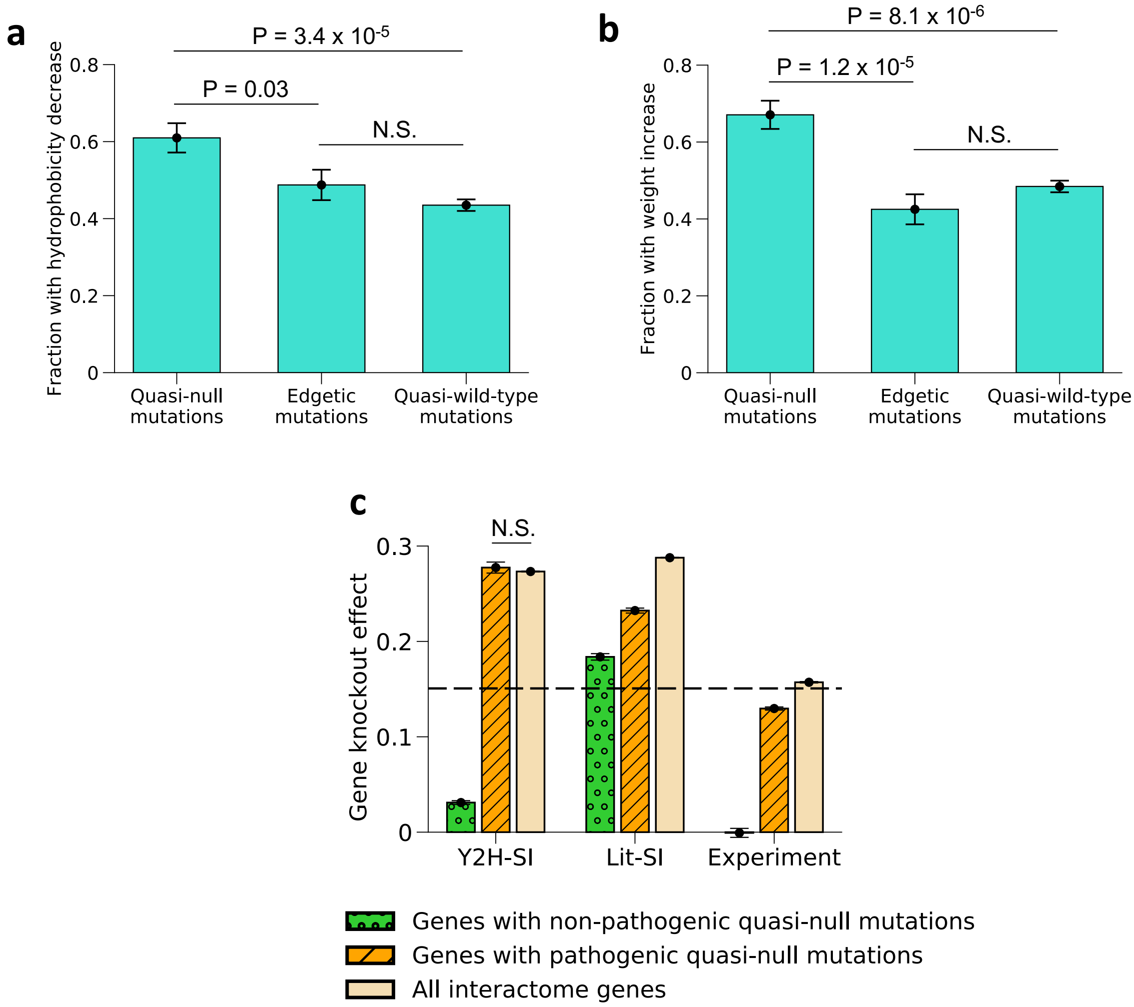

Figure S5 Biophysical properties of different mutation edgotype classes

(a) Fraction of pathogenic mutations within each edgotype class identified by predictions and experiments that involve a decrease in residue hydrophobicity upon mutation. **(b)** Fraction of pathogenic mutations within each edgotype class identified by predictions and experiments that involve an increase in residue molecular weight upon mutation. Error bars in (a) and (b) represent standard errors of the fraction. P-values in (a) and (b) were calculated using a two-sided Fisher's exact test. **(c)** Gene knockout effect in 808 cancer cell lines obtained from the DepMap project for genes encoding proteins that are disrupted by pathogenic or non-pathogenic quasi-null mutations in the two human structural interactomes, Y2H-SI and Lit-SI, and in the experimental dataset of Sahni et al. (2015). Higher values indicate more detrimental effects. Dashed horizontal line represents the genome average. Error bars represent standard errors of the mean. P-value < 10^{-29} for all adjacent bars within each group except those labeled with N.S. (not significant). P-values were calculated using a two-sided t-test.

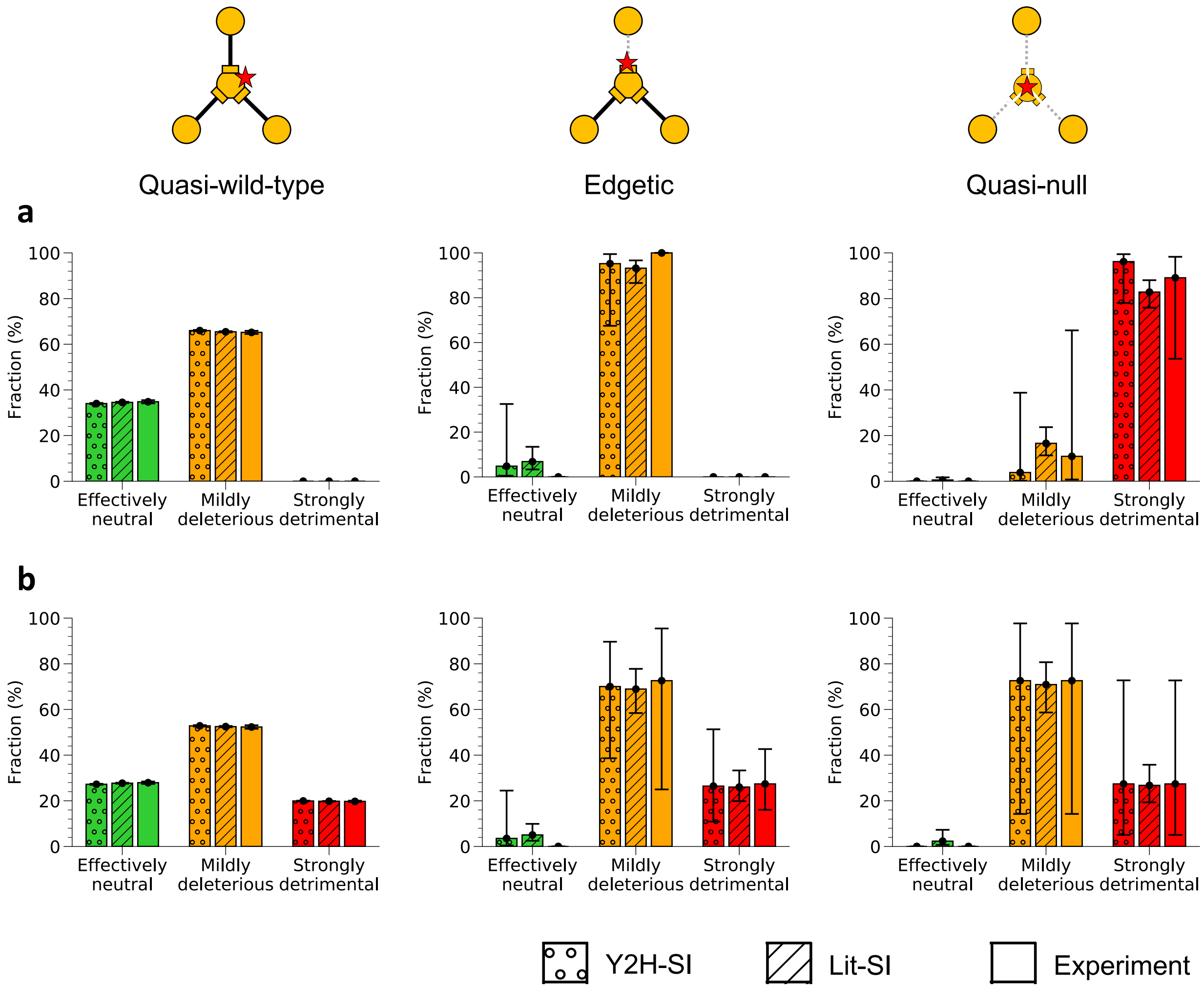

Figure S6 Edgotype fitness effect among proteins encoded by essential genes
Probabilities for quasi-wild-type mutations (left), edgetic mutations (middle), and quasi-null mutations (right) among proteins encoded by essential genes to be effectively neutral, mildly deleterious, or strongly detrimental in human. Essential genes are those found to be commonly essential among all cancer cell lines in the DepMap project. Probabilities were estimated from mutation edgotypes obtained by structure-based predictions in the two human structural interactomes, Y2H-SI and Lit-SI, and from mutation edgotypes obtained by experiments. **(a)** Fitness effect calculated assuming that strongly detrimental mutations are all quasi-null (Assumption I), with a fraction of them disrupting proteins encoded by essential genes equal to the fraction of essential genes in each interactome **(b)** Fitness effect calculated assuming that strongly detrimental mutations are as likely as mildly deleterious mutations to be quasi-wild-type, edgetic or quasi-null among proteins encoded by essential genes (Assumption II). Error bars represent 95% confidence intervals.
